# Supplementary material for: Multiome-based identification of molecular markers for prospective identification of platelet-biased HSCs
Source: Stem Cell Reports. 2026 Jun 11;21(7):102959. doi: 10.1016/j.stemcr.2026.102959 (PMC13385442; doi:10.1016/j.stemcr.2026.102959)
Supplement: Document S1. Figures S1–S7 [file mmc1.pdf]

Stem Cell Reports, Volume *21*

Supplemental Information

Multiome-based identification of molecular markers for prospective identification of platelet-biased HSCs

Bowen Zhang, Yiran Meng, Esther Rodríguez Correa, Xiying Ren, Alexandre Fagnan, Michael D. Milsom, and Claus Nerlov

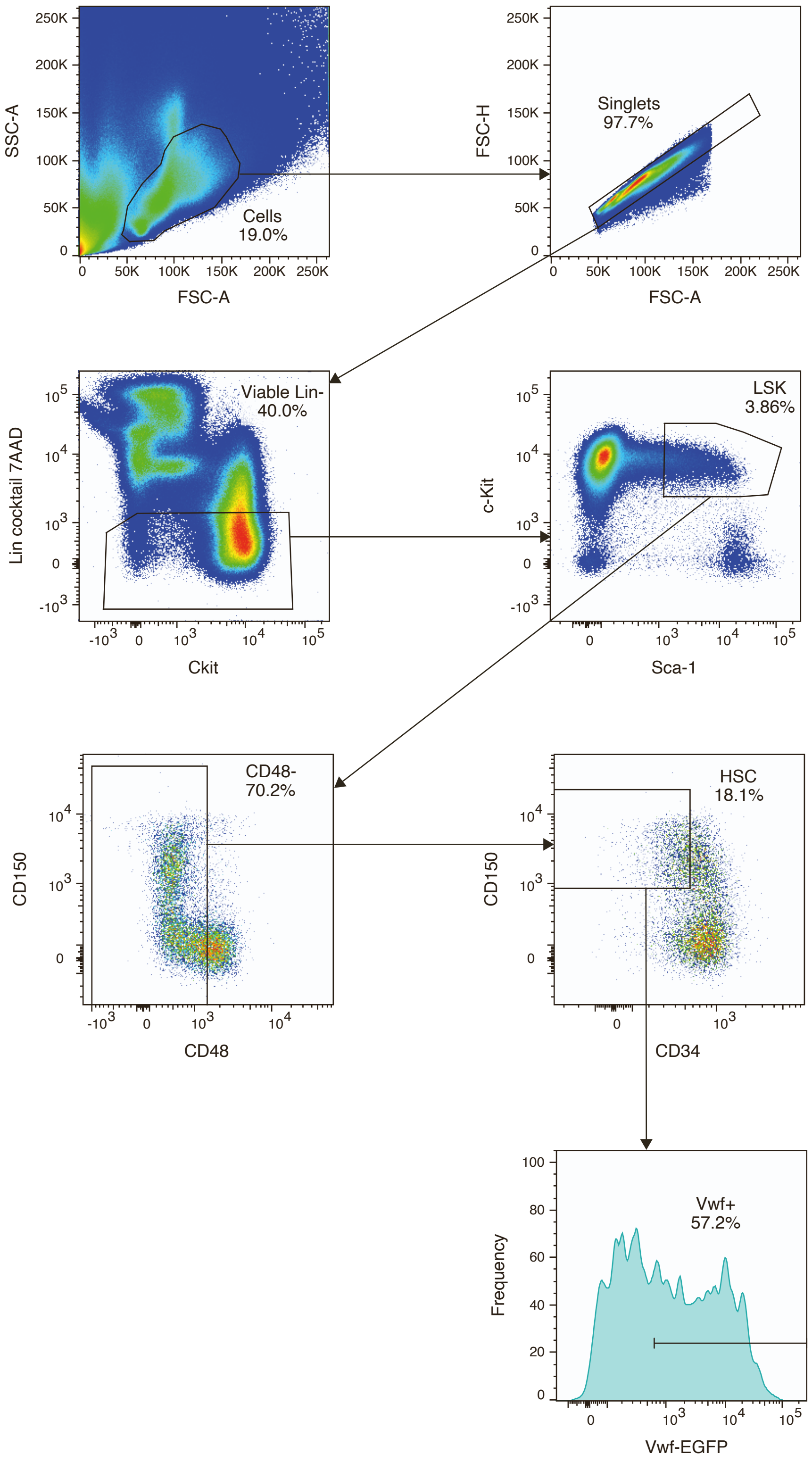

Supplementary Figure 1

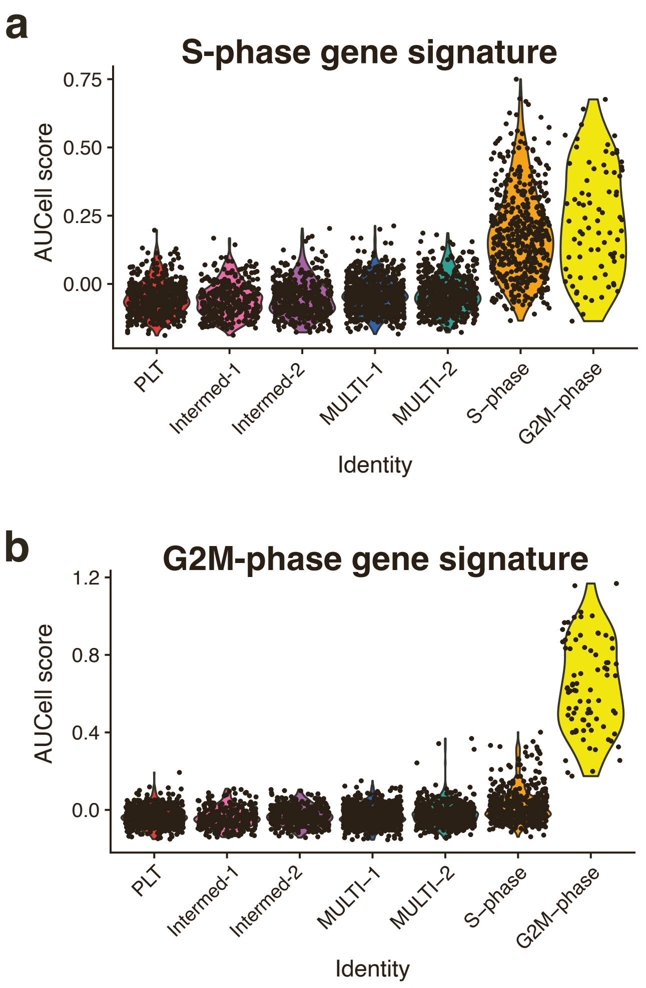

Supplementary Figure 2

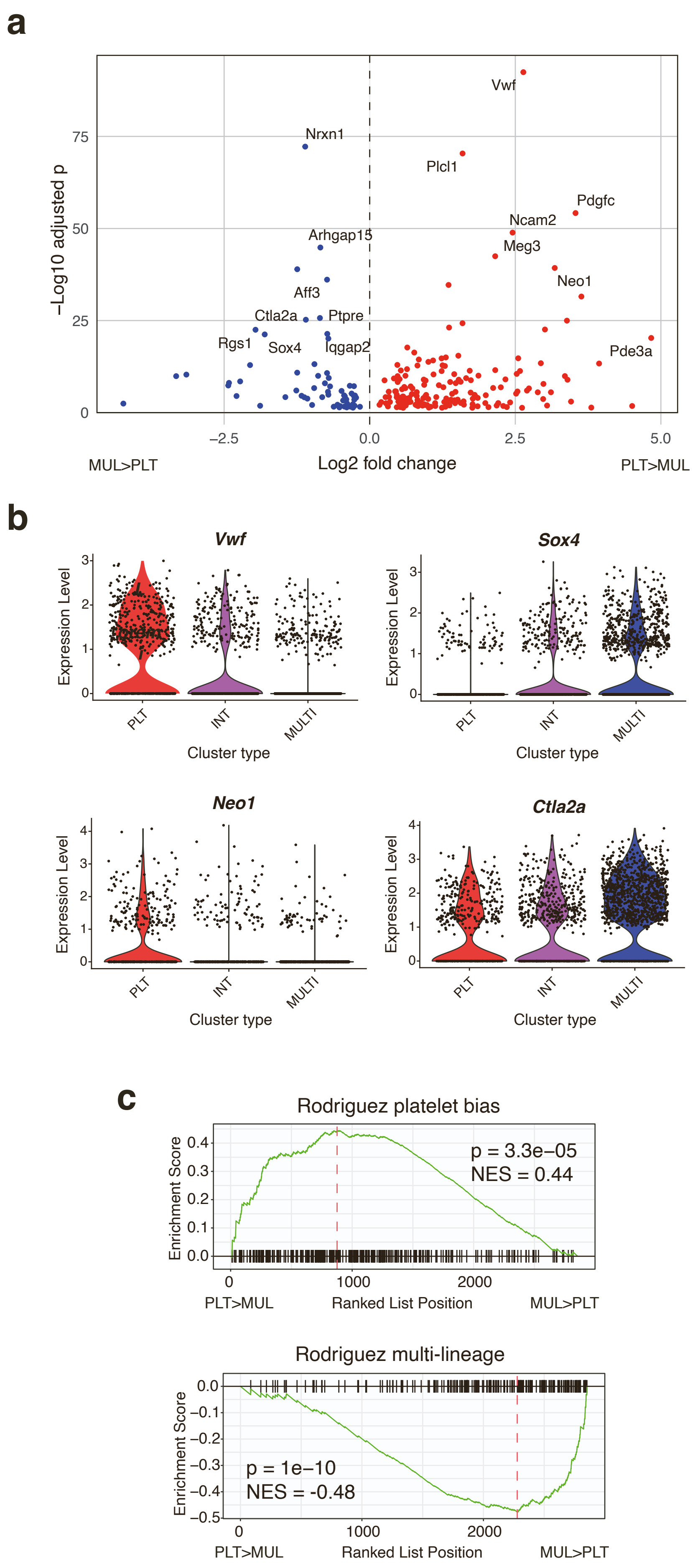

Supplementary Figure 3

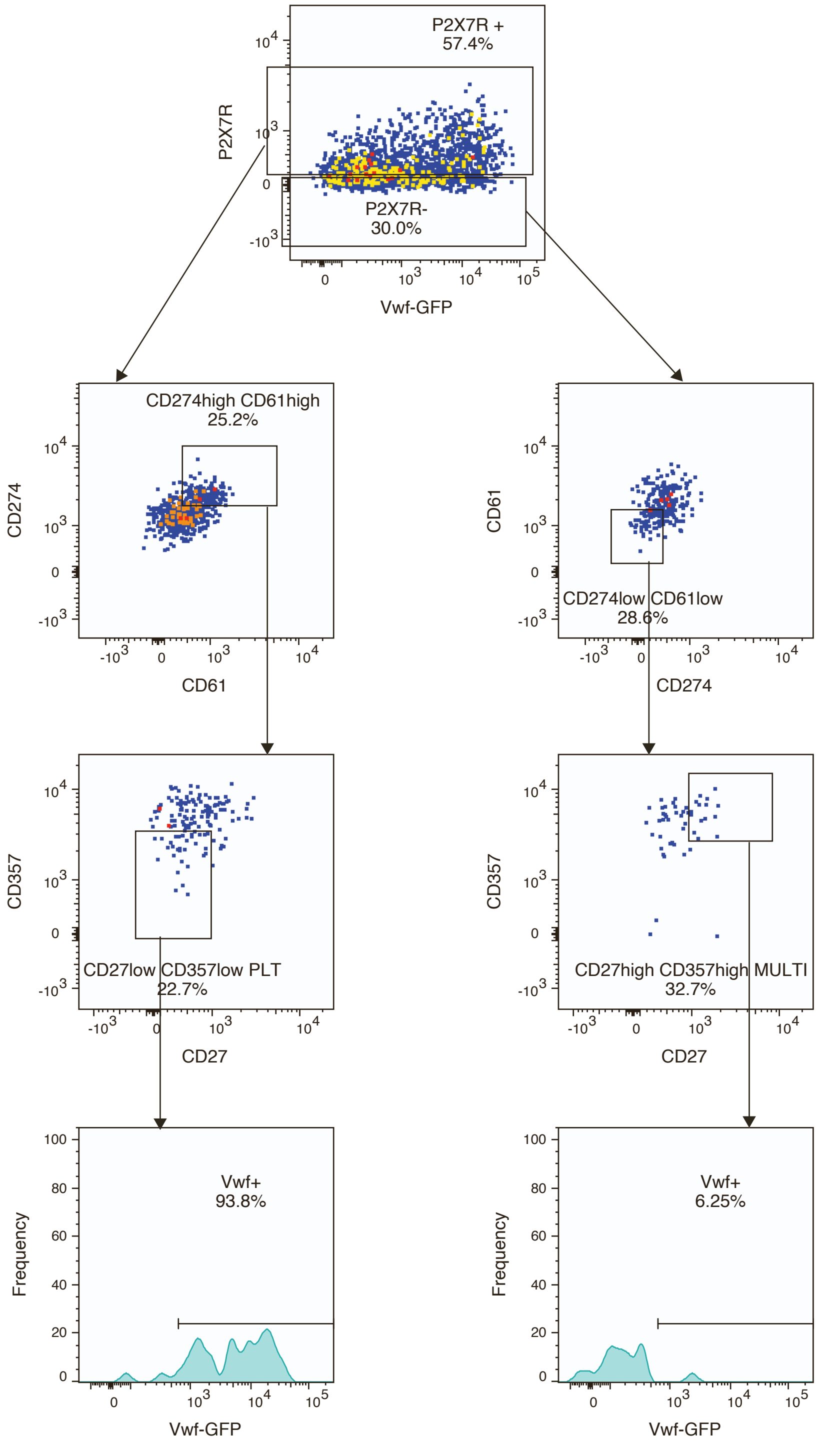

Supplementary Figure 4

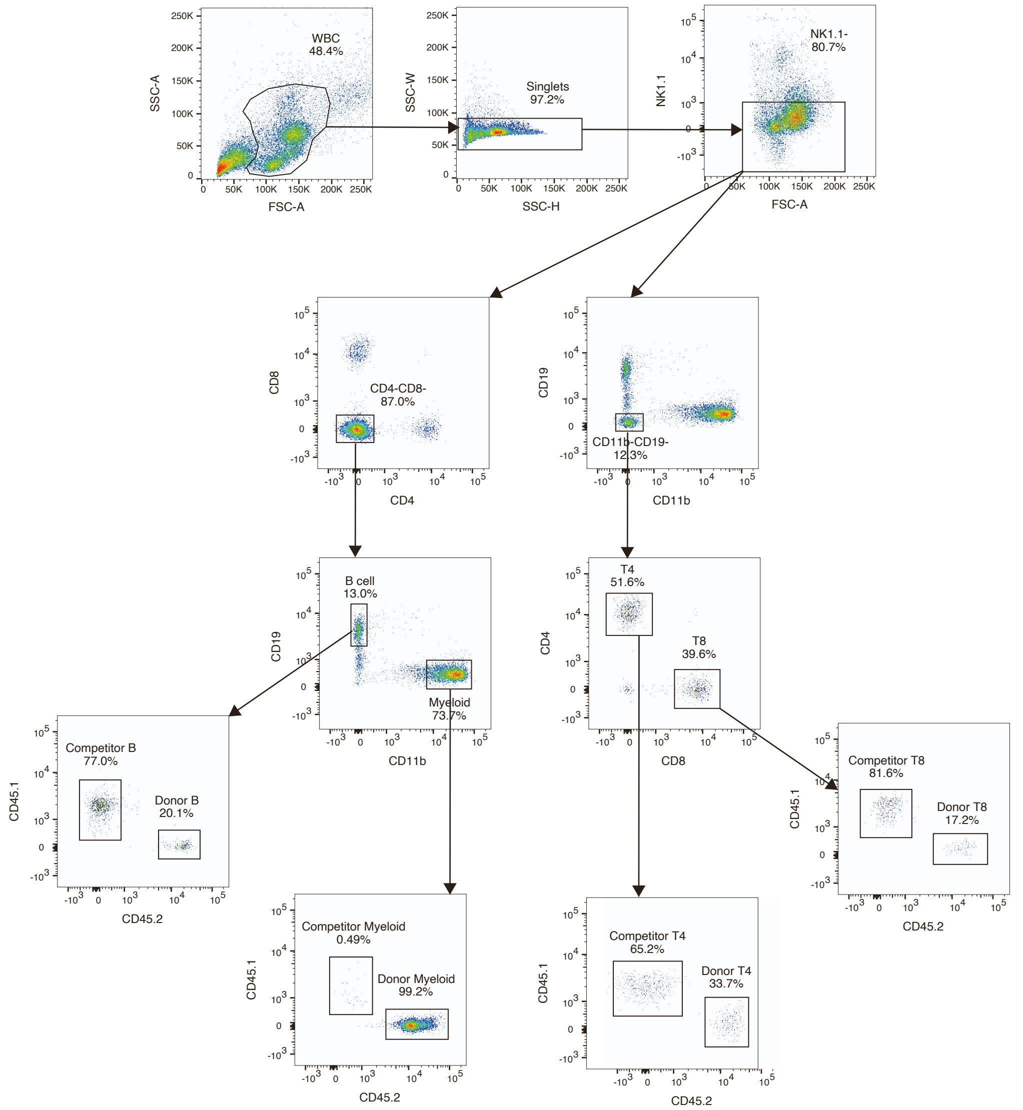

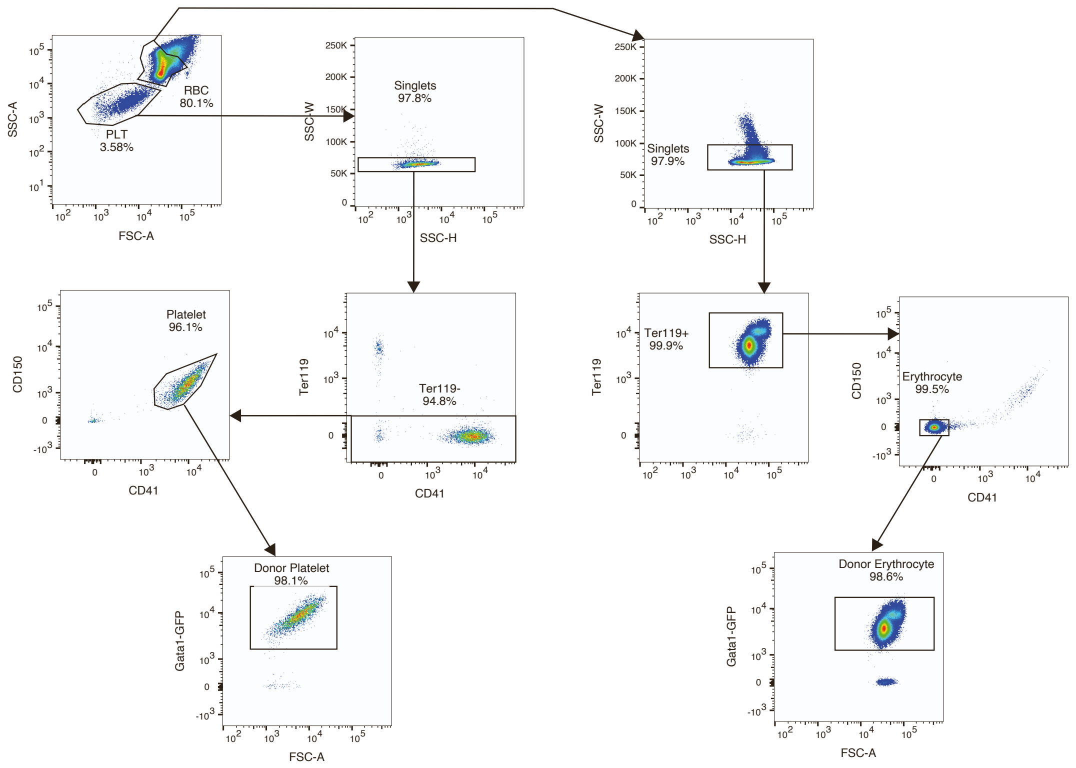

Supplementary Figure 5

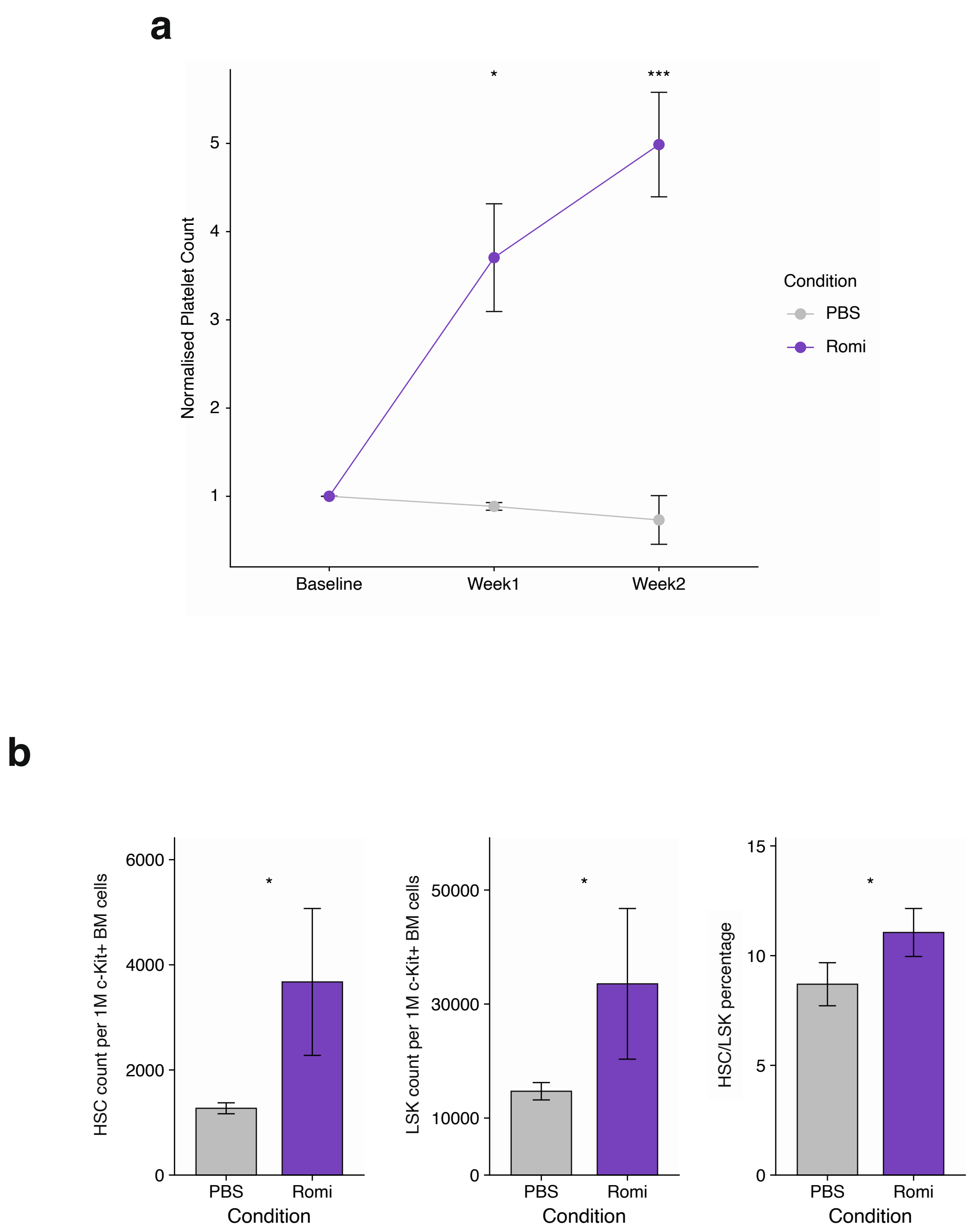

Supplementary Figure 6

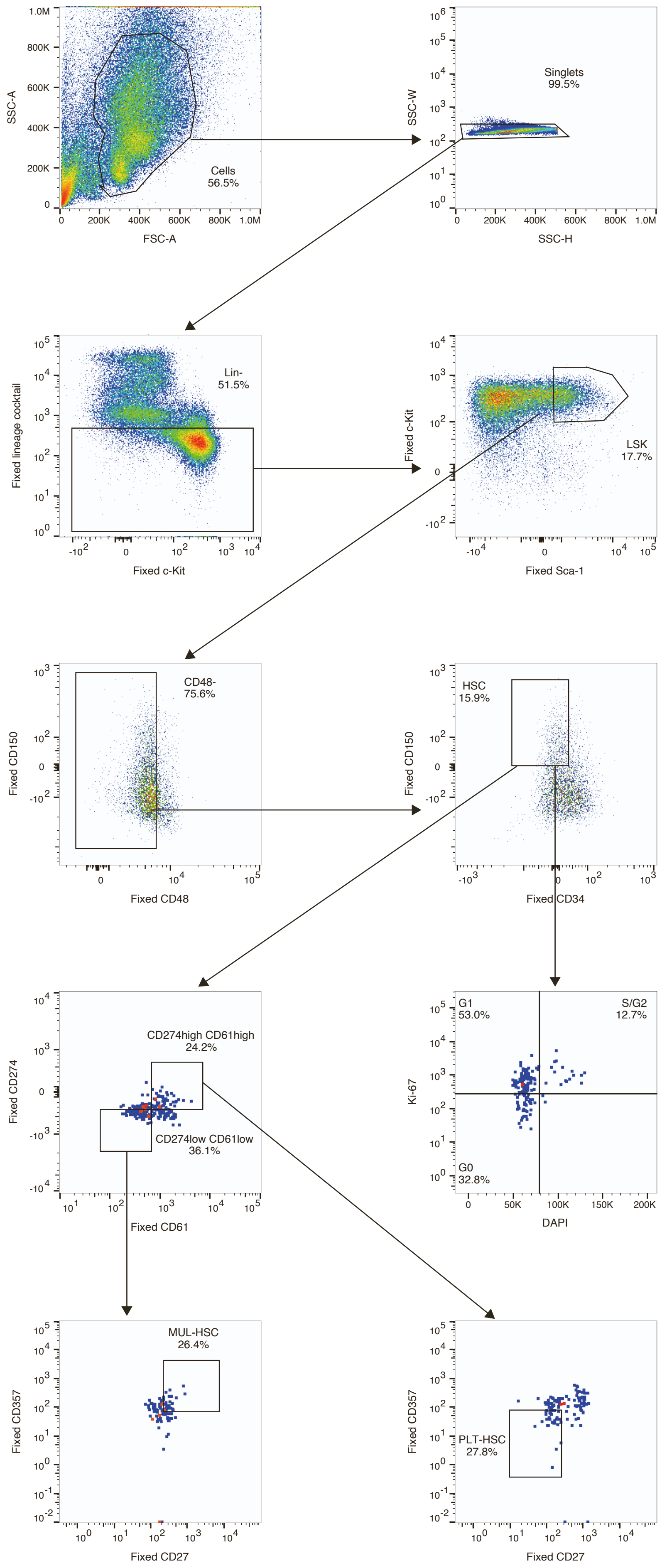

Supplementary Figure 7

Supplementary Figure legends.

Supplementary Figure 1 | Gating strategy for identifying mouse HSCs

Flow cytometry gating strategy for identifying HSCs (LSKCD150+CD48–CD34–) from *Vwf*-EGFP transgenic mice. The percentage of *Vwf*-EGFP positive cells in the total HSC population is shown. This strategy was also used for purifying HSCs from WT and *Gata1*-EGFP transgenic mice.

Supplementary Figure 2 | Cell cycling scores of 8-week HSCs.

A) Gene expression-inferred S phase score of clusters from Figure 2A.

B) Gene expression-inferred G2M phase score of clusters from Figure 2A.

Supplementary Figure 3 | Annotation of non-cycling WNN clusters

A) Volcano plot of genes differentially expressed ($P<0.05$) between PLT and MUL clusters in HSCs from Figure 2A.

B) Expression levels of selected lineage potential signature genes in the PLT, INT and MUL clusters from Figure 2A.

C) GSEA analysis comparing the PLT and MUL clusters using previously defined HSC lineage bias signatures for platelet-biased and multi-lineage HSCs.

Statistical comparisons performed using MAST for single-cell differential gene analysis and permutation test for GSEA enrichment.

Supplementary Figure 4 | FACS enrichment for *Vwf*+ HSCs with the five-marker strategy

Flow cytometry gating strategy using the top five RFE-ranked markers from Figure 2E. The percentage of *Vwf*-EGFP positive cells is shown in PLT- (LSKCD150+CD48–CD34–P2X7R+CD61^{hi}CD274^{hi}CD357^{lo}CD27^{lo}) and MUL-HSCs (LSKCD150+CD48–CD34–P2X7R-CD61^{lo}CD274^{lo}CD357^{hi}CD27^{hi}) from *Vwf*-EGFP transgenic mice.

Supplementary Figure 5 | Peripheral blood lineage reconstitution analysis in mice transplanted with single HSCs

A) Flow cytometry gating strategy for identifying donor-derived CD45.1 myeloid cells, B cells and T cells.

B) Flow cytometry gating strategy for identifying donor-derived *Gata1*-EGFP+ platelets and erythrocytes.

Supplementary Figure 6 | Peripheral blood and endpoint bone marrow analysis in mice treated with Romiplostim

A) Normalised peripheral blood platelet count after Romiplostim (Romi) treatment or control.

B) LSK and HSC (LSKCD150+CD48–CD34–) cell counts per million c-Kit-enriched bone marrow cells and the HSC/LSK ratio.

Mouse number: Romi, N=4; PBS, N=4. Statistical comparisons performed using unpaired two-tailed T-tests; *$P < 0.05$, ***$P < 0.001$. Data are presented as mean ± standard deviation.

Supplementary Figure 7 | Gating strategy for fixed mouse bone marrow

Flow cytometry gating strategy for identifying PLT- and MUL-HSC, and assessing cell cycle status in permeabilized mouse bone marrow samples. Cell cycle phases are defined as G0 ($DAPI^{lo}Ki\text{-}67^{lo}$), G1 ($DAPI^{lo}Ki\text{-}67^{hi}$) and S/G2/M ($DAPI^{hi}Ki\text{-}67^{hi}$)

Supplementary Table legends.

Table S1 | Differentially expressed genes between PLT and MUL-HSC clusters from 8-week-old mice.

Differential expression analysis was performed using MAST, with a significance threshold of $P < 0.05$.

Table S2 | Differentially expressed genes between clonal PLT and MUL-HSC populations.

Differential expression analysis was performed using DESeq2, with a significance threshold of $P < 0.0005$. The max_tpm column reports the highest transcript per million (TPM) values

detected across all clones. The var_PLT and var_MUL columns represent the maximum variation in TPM between biological replicates within the PLT and MUL clones, respectively.

Table S3 | Antibody-derived tags.

The titration column indicates the titration factor relative to the corresponding unconjugated antibody.

Table S4 | Oligos.

Shows the sequence of pre-amplification, sample index PCR primers for constructing DOGMAseq libraries, and the antibody derived tag oligo.

Table S5 | TaqMan assays for microfluidics-based qRT-PCR.

This panel allows accurate phenotyping of lineage potential in mouse HSCs.

Table S6 | Normalized gene expression values from microfluidics-based qRT-PCR of HSC populations.

Shows the integrated lineage potential signature gene expression profiles used in (Figure 5D).

Table S7 | FACS antibodies.

List of antibodies used for flow cytometry and cell sorting.
