# Supplementary material for: Multiome-based identification of molecular markers for prospective identification of platelet-biased HSCs
Source: Stem Cell Reports. 2026 Jun 11;21(7):102959. doi: 10.1016/j.stemcr.2026.102959 (PMC13385442; doi:10.1016/j.stemcr.2026.102959)
Supplement: Document S2. Article plus supplemental information [file mmc3.pdf]

STEM CELL REPORTS
Article

OPEN ACCESS

Multiome-based identification of molecular markers for prospective identification of platelet-biased HSCs

Bowen Zhang,[1] Yiran Meng,[1] Esther Rodríguez Correa,[2] Xiying Ren,[1] Alexandre Fagnan,[1] Michael D. Milsom,[2] and Claus Nerlov[1,3,*]

[1]MRC Molecular Haematology Unit, MRC Weatherall Institute of Molecular Medicine, University of Oxford, John Radcliffe Hospital, Headington, Oxford, UK

[2]Heidelberg Institute for Stem Cell Technology, Im Neuenheimer Feld 280, 69120 Heidelberg, Germany

[3]Lead contact

*Correspondence: claus.nerlov@imm.ox.ac.uk

https://doi.org/10.1016/j.stemcr.2026.102959

SUMMARY

Platelet-biased hematopoietic stem cells (PLT-HSCs) play key roles in normal physiology, aging, and blood cancer. However, currently, no markers allow their accurate identification or prospective isolation. We here combine single-mouse hematopoietic stem cell (HSC) gene expression, chromatin accessibility, and surface proteome profiling to identify subtype-specific markers. Using machine learning, we identified markers ($CD61^{hi}CD274^{hi}CD357^{lo}CD27^{lo}$) that isolate PLT-HSCs to high purity, validated by single-cell transplantation. Furthermore, we develop a minimal expression marker panel that discriminates PLT- and multi-lineage (MUL-)HSCs using microfluidics-based single-cell RT-qPCR. We show that both methods detect the age-associated increase in PLT-HSCs, while poly(I-C)-induced chronic inflammation did not alter HSC lineage bias. In contrast, romiplostim treatment increased MUL-HSC prevalence. Finally, using spectral flow cytometry to simultaneously quantify cell cycle and HSC lineage bias, we show that platelet depletion selectively activates PLT-HSCs. Together, these approaches allow accurate isolation of PLT-HSCs and robust quantification of lineage bias under perturbation.

INTRODUCTION

Hematopoietic stem cells (HSCs) sustain blood cell production throughout the mammalian lifespan and are the central cell type in bone marrow transplantation, the most widely used cell therapy. HSC populations are functionally heterogeneous, with single-cell transplantation showing that clonal HSC long-term reconstitution can generate both myeloid and lymphoid lineages at similar levels (hereafter referred to as multi-lineage- or MUL-HSCs) or lack lymphoid output and show bias toward platelet-myeloid output (platelet-biased or PLT-HSCs) (Meng and Nerlov, 2025). PLT-HSCs were first identified based on their expression of a *Vwf*-EGFP transgene (Sanjuan-Pla et al., 2013), and this transgene has been used to show selective expansion of PLT-HSCs during physiological aging (Grover et al., 2016), to identify specific PLT-HSC niches (Pinho et al., 2018), and to show that PLT-HSCs initiate JAK2-induced myeloproliferation (Kimmerlin et al., 2024). However, while *Vwf*-EGFP+ HSCs have enhanced platelet output compared to *Vwf*-EGFP− HSCs (Sanjuan-Pla et al., 2013), subsequent single-cell transplantations showed that the *Vwf*+ HSC fraction was composed of roughly equal proportions of PLT- and MUL-HSCs, whereas *Vwf*− HSCs were composed of MUL- and lymphoid-biased (L)-HSCs (Carrelha et al., 2018). This, along with the absence of other specific PLT-HSC markers, significantly limits our ability to accurately identify PLT-HSCs using simple molecular markers, by imaging or with flow cytometry, and to prospectively isolate them by cell sorting. Consequently, molecular characterization of HSC subtypes has relied on cellular barcoding combined with single-cell transcriptome analysis (Pei et al., 2020; Rodriguez-Fraticelli et al., 2020) and profiling of clonal HSC populations (Meng et al., 2023), where PLT-HSCs can be retrospectively identified based on their lineage output or using markers such as *Vwf*-EGFP that are only moderately selective. To address this knowledge gap, we here profile single HSCs by simultaneous RNA sequencing (RNA-seq), assay for transposase-accessible chromatin (ATAC) using sequencing (ATAC-seq), and CITE-seq, using antibodies against antigens that are predicted to be differentially expressed across HSC subtypes. This allowed the identification of genes and surface markers expressed preferentially by PLT-HSCs (CD274 and CD61) and MUL-HSCs (CD27 and CD357). We show that combining these markers allows PLT-HSCs to be prospectively identified and isolated to ~85% purity from young adult mice, as measured by single-cell transplantation. Furthermore, we use this dataset to develop a simple gene expression profile that can quantify platelet bias at the single-HSC level. Finally, we use these two platforms to show that aging and acute platelet depletion, but not chronic inflammatory stress, generate platelet bias of the HSC population.

RESULTS

Identification of surface markers selective for HSC subtypes

To identify putative surface markers specific to HSC subtypes, we used previously generated gene expression profiles obtained from HSC clones with defined fate restriction

Published by Elsevier Inc. on behalf of International Society for Stem Cell Research.
This is an open access article under the CC BY license (http://creativecommons.org/licenses/by/4.0/).

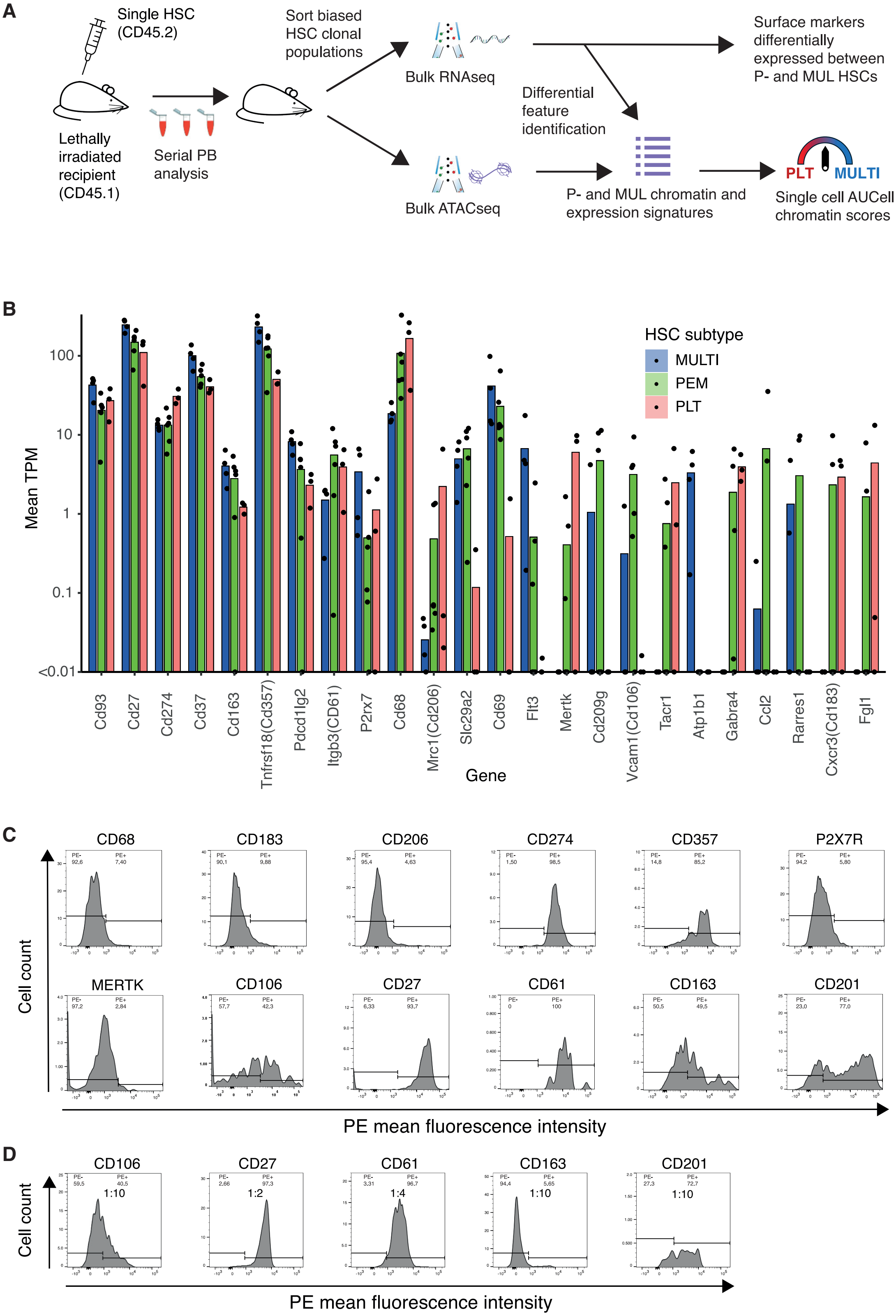

(legend on next page)

(Meng et al., 2023). These clones were obtained by transplantation of single *Vwf*-tdTomato+ HSCs and classified by regular analysis of their lineage output over 4 months (Figure 1A). DESeq2 was used to compare PLT-HSCs, MUL-HSCs, and platelet-erythroid-myeloid (PEM)-HSCs, and differentially expressed genes were filtered for cell surface expression and the availability of antibodies. This yielded 24 putative marker genes for downstream analysis (Figure 1B). We stained HSCs (defined as Lin−Sca-1+c-Kit+CD150+CD48−CD34−; Figure S1) from *Vwf*-EGFP transgenic mice (Sanjuan-Pla et al., 2013) with PE-conjugated antibodies against the candidate markers and observed significant signal from 12 of these antibodies (Figure 1C).

To correlate surface marker expression to HSC fate restriction, we used DOGMA-seq (Mimitou et al., 2021), which allows simultaneous measurement of transcriptome, chromatin accessibility, and antibody surface binding at the single-cell level. In order to balance the detection of antibodies during sequencing, we first established suitable dilutions of antibodies with high staining intensity on HSCs (Figure 1D). Using this optimized antibody panel of oligonucleotide-conjugated antibodies, we stained and purified HSCs from 8-week-old mice (using the gating strategy from Figure S1) followed by 10× multiome profiling using the DOGMA-seq protocol. After mapping of reads to the 3 modalities, single HSCs were clustered using weighted nearest neighbor (WNN) integration of RNA-seq and ATAC-seq (Figure 2A). Clusters representing cycling HSCs were identified using transcriptional cell cycle signatures (Figures S2A and S2B). To assign HSC subtype identity to non-cycling HSC clusters, we used ATAC signatures specific for MUL- and PLT-HSCs generated from the aforementioned fate-restricted HSC clones (Meng et al., 2023). Based on the PLT/MUL ATAC signature ratio, clusters were classified as PLT-HSCs (high signature ratio), MUL-HSCs (low signature ratio), or INT-HSCs (intermediate signature ratio), likely representing PEM-HSCs (Meng et al., 2023) (Figure 2B). This molecular classification was further supported by transcription factor (TF) motif enrichment analysis. Comparison of PLT, MUL, and INT clusters revealed enrichment of previously identified MUL-specific motifs in MUL-HSCs and PLT-specific motifs in PLT-HSCs, whereas the INT clusters exhibited the characteristic intermediate TF motif accessibility profile of PEM-HSCs (Figure 2C). Finally, differential gene expression analysis comparing PLT- and MUL-HSC clusters (Figure S3A; Table S1) revealed known marker genes preferentially expressed in PLT-HSCs (*Vwf* and *Neo1*) and MUL-HSCs (*Ctla2a* and *Sox4*) (Figure S3B). Consistently, gene set enrichment analysis (GSEA) confirmed enrichment of previously defined transcriptional PLT- and MUL-HSC signatures in PLT and MUL clusters, respectively (Rodriguez-Fraticelli et al., 2020) (Figure S3C).

Using the molecularly annotated clusters, antibody tag expression on HSC subtypes was quantified (Figure 2D), and recursive feature elimination (RFE) was used to rank CITE-seq markers by their ability to discriminate PLT- and MUL-HSC clusters (Figure 2E). *In silico* gating using the top-ranked markers enriched for PLT-HSCs ($CD61^{hi}CD274^{hi}$ $CD357^{lo}CD27^{lo}P2X7R^{lo}$) or, conversely, MUL-HSCs ($CD61^{lo}$ $CD274^{lo}CD357^{hi}CD27^{hi}P2X7R^{hi}$) (Figures 2F and 2G). As the INT clusters have molecular properties of PEM-HSCs (which are platelet biased), this analysis indicates an approximate 75% enrichment PLT-HSCs using the PLT gating and similar 75% enrichment of MUL-HSCs using the MUL gating, excluding cycling HSCs from the calculation.

Validation of marker-defined HSC subsets

The identified markers therefore enrich HSC subsets as measured by *in silico* gating. To assess their ability to fractionate HSCs *in vivo*, we first assessed the ability of the identified markers to purify HSCs based on expression of the *Vwf*-EGFP reporter (Sanjuan-Pla et al., 2013). We previously found that this *Vwf* reporter was expressed in the vast majority of PLT-HSCs (93% ± 4%) but only in a small minority of MUL-HSCs (7% ± 4%) (Carrelha et al., 2018). Using the 5-marker panel to gate PLT- and MUL-HSCs from *Vwf*-EGFP transgenic mice, we found these to have the expected proportions of *Vwf*-EGFP+ cells (Figure S4). Elimination of individual markers showed that P2XR7 contributed minimally to the efficiency of the marker panel (Figure 3A),

Figure 1. Development of the CITE-seq antibody panel

(A) Experimental workflow for profiling lineage-restricted HSC subtypes. Single HSCs (LSKCD150+CD48−CD34−*Vwf*+) were transplanted into lethally irradiated recipient mice along 200,000 competitor bone marrow cells. Serial peripheral blood analysis identified lineage-restricted donor cells. Clonal HSC populations were re-isolated for bulk RNA-seq and ATAC-seq. The differential gene expression and accessible chromatin signatures were utilized for scoring molecular lineage bias of individual HSCs. The differentially expressed genes from RNA-seq analysis were used to identify candidate subtype-specific cell-surface markers for CITE-seq.

(B) Mean transcripts per million (TPM) values of CITE-seq candidate genes across clonally amplified HSC subtypes from (A). Number of independent single-HSC-derived clones: PLT-HSCs, $N = 3$; PEM HSCs, $N = 6$; MUL-HSCs, $N = 4$.

(C) PE antibody staining intensity of the proteins encoded by CITE-seq candidate genes from (B) on mouse HSCs.

(D) Candidate markers with high staining intensities in (C) were titrated with their unconjugated forms to standardize staining levels across the panel. These titration ratios shown were used in the final CITE-seq antibody panel to ensure balanced detection of antigens.

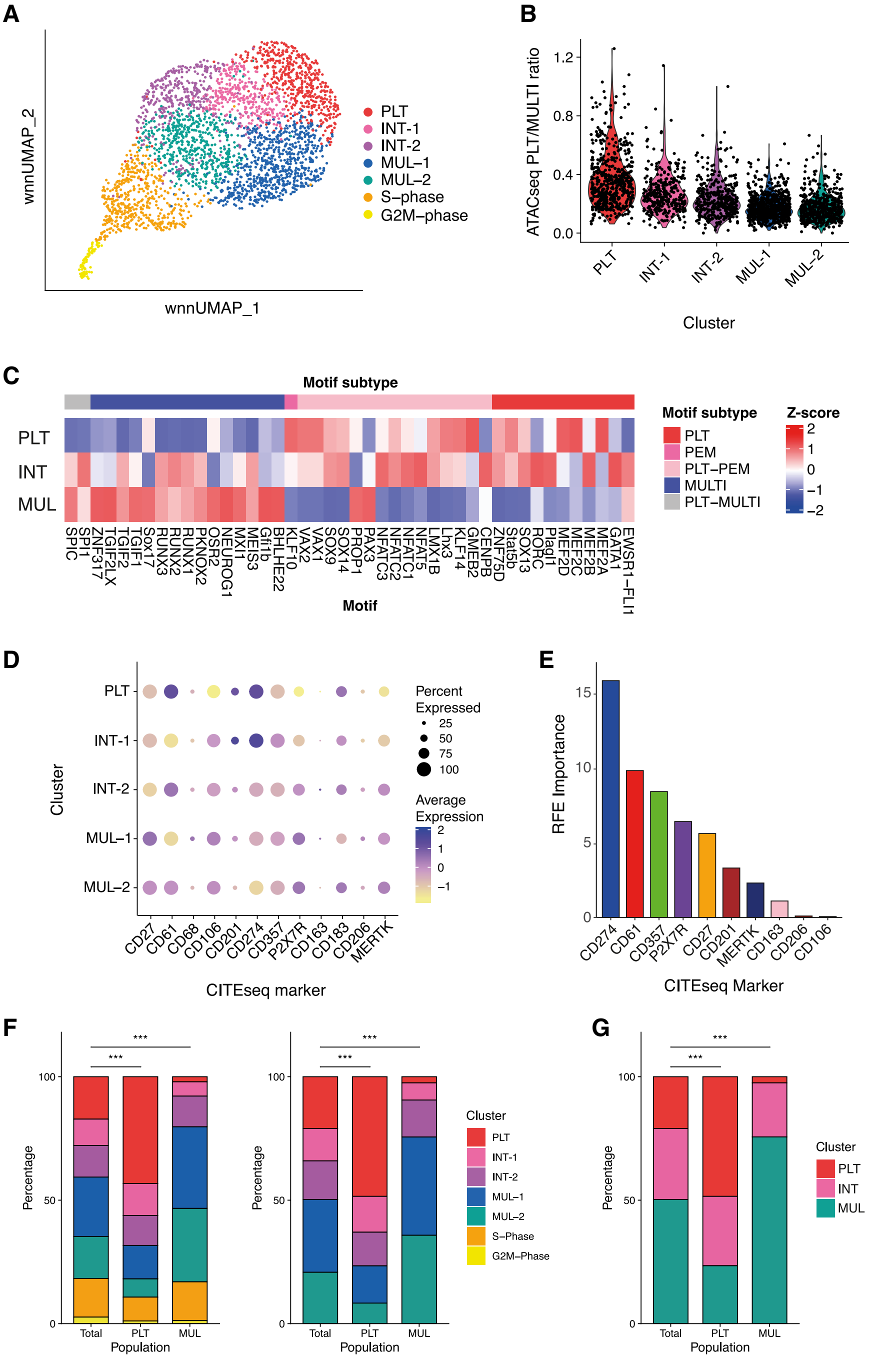

Figure 2. Identification of CITE-seq markers

(A) Weighted nearest neighbor (WNN) uniform manifold approximation and projection (UMAP) projection of HSCs from 8-week-old mice. Clusters were annotated by their HSC subtype signature scores and cell cycling status.

(B) Per-cell HSC subtype signature scores derived from ATAC-seq signatures in the non-cycling clusters from (A).

(C) Heatmap of average accessibility scores (ChromVAR) of transcription factor motifs specific for the indicated HSC subtypes in HSC clusters from (B). INT and MUL clusters have been combined.

(D) Expression of CITE-seq markers in clusters from (B). Color intensity indicates scaled expression; dot size represents the fraction of expressing cells.

(E) Importance scores of CITE-seq markers for distinguishing PLT and MUL clusters, determined by recursive feature elimination (RFE).

(F) *In silico* gating simulation of HSCs from (A) using the top five RFE-ranked markers from (E). The proportion of total, PLT- and MUL-gated cells belonging to each cluster from (A) is shown, including (left) and excluding (right) the cycling clusters.

(G) HSC labels from (F) were grouped into PLT, INT (INT-1 and INT-2 combined), and MUL (MUL-1 and MUL-2 combined) populations.

Statistical comparisons performed using chi-squared tests for *in silico* gating; $***p < 0.001$.

and the resulting 4-marker panel was used for further experiments. To further validate the gating strategy, we generated a panel of genes preferentially expressed in PLT- and MUL-HSCs, respectively (Figure 3B; Table S2). PLT- and MUL-HSCs were sorted as outlined in Figure 3A, and gene expression was profiled by microfluidics-based single-cell PCR followed by principal-component analysis (PCA) (Figure 3C). This showed that the 4-marker panel efficiently segregated PLT- and MUL-HSCs defined by gene expression (Figure 3D). Finally, to functionally validate the PLT-HSC markers, we performed single-HSC transplantation. HSCs were sorted from CD45.1 *Gata1*-EGFP transgenic mice to allow quantification of all lineages, including platelets and erythrocytes, after transplantation into CD45.2 recipients. Lineage bias of HSC clones was determined by quantification of lineage output after 6, 10, and

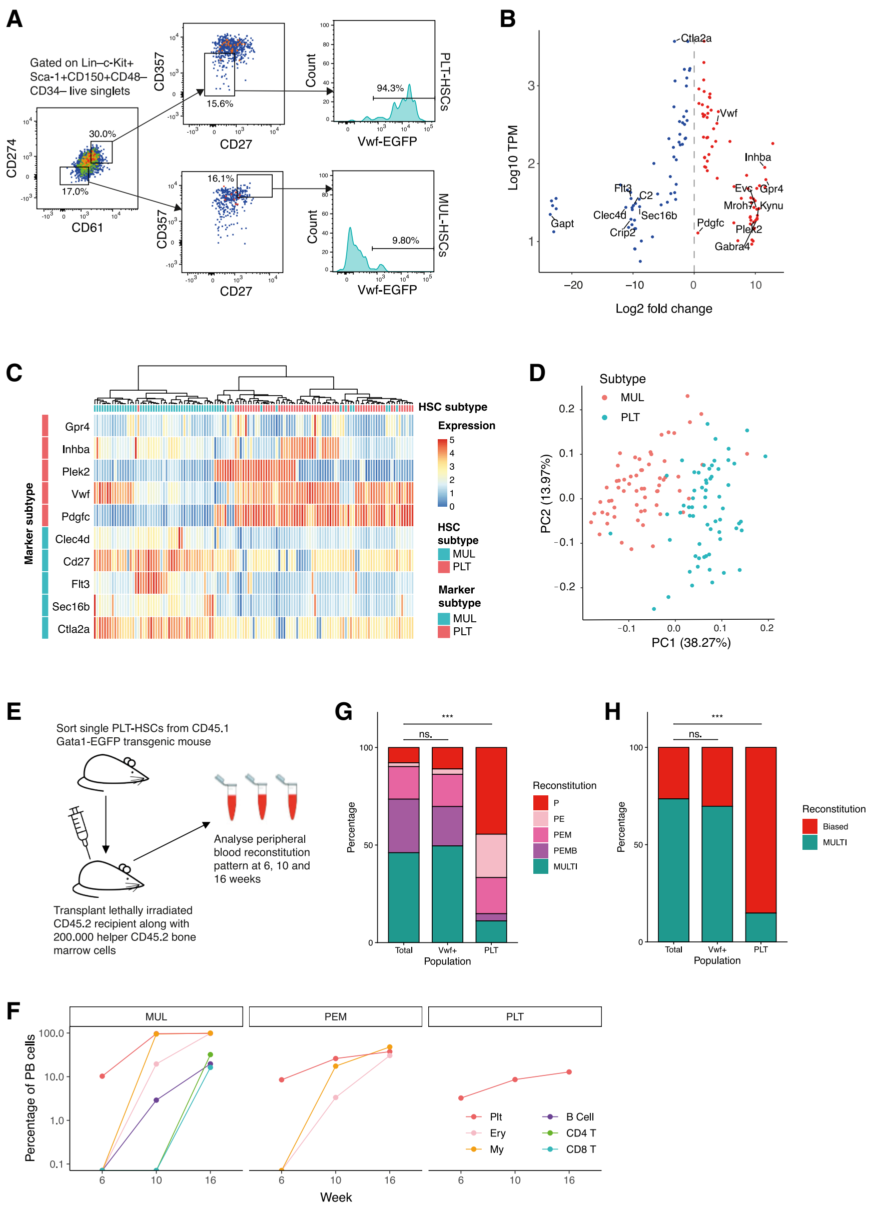

Figure 3. FACS isolation using new markers enriches lineage-biased HSCs

(A) Flow cytometry gating strategy for identifying PLT- (LSKCD150+CD48−CD34−CD61^{hi}CD274^{hi}CD357^{lo}CD27^{lo}) and MUL-HSCs (LSKCD150+CD48−CD34−CD61^{lo}CD274^{lo}CD357^{hi}CD27^{hi}) from *Vwf*-EGFP transgenic mice. The percentage of *Vwf*-EGFP-positive cells in the PLT- and MUL-HSC populations is shown.

(B) Volcano plot of genes differentially expressed ($p < 0.05$) between clonal PLT and MUL-HSC populations from Figure 1B.

(C) Heatmap of gene expression in single PLT- and MUL-HSCs sorted as in (A) and profiled using microfluidics-based RT-qPCR of the indicated subtype-specific genes. Cells were hierarchically clustered using the normalized gene expression values. Mouse number: $N = 4$.

(D) Plot of the PLT- and MUL-HSCs from (C) using the first two components from principal-component (PCA) analysis of their gene expression. Note the efficient separation of HSC subtypes.

(E) Experimental workflow for single-cell transplantation of purified PLT-HSCs. Single PLT-HSCs were isolated from 8- to 12-week-old *Gata1*-EGFP mice (CD45.1 allotype) and transplanted into lethally irradiated CD45.2 recipient mice along 200,000 CD45.2 competitor bone marrow cells. Lineage output from single HSCs was assessed by serial peripheral blood analysis.

(F) Examples of peripheral blood reconstitution patterns of MUL-, PEM-, and PLT-HSC clones from single HSC transplanted as shown in (E).

(G) Distribution of lineage reconstitution patterns in PLT (CD61^{hi}CD274^{hi}CD357^{lo}CD27^{lo}), *Vwf*+, and total HSCs.

(H) HSCs from (G) were grouped into platelet-biased (P-bias, P; PE or PEM-restricted) and multi-lineage HSCs (multi-PEMB and PEMBT) HSC phenotypes.

Number of transplant recipients: PLT-HSC, $N = 27$; *Vwf*+ HSC, $N = 102$; total HSC, $N = 109$. Statistical comparisons performed using DEseq2 Wald's test for differential gene analysis, chi-squared test for transplanted HSCs; $^{***}p < 0.001$.

16 week (Figures 3E, 3F, and S5). We observed the same output patterns as previously described, including a progressively lower contribution to overall platelet production with increasing platelet restriction (Carrelha et al., 2018). Using the 4-marker panel, we were able to obtain 85% purity of PLT-HSCs, defined as HSCs lacking lymphoid output and where platelets were the most abundant output lineage, compared to the previously observed 26% of total HSCs and 30% of *Vwf*+ HSCs (Carrelha et al., 2018) (Figures 3G and 3H). The majority of PLT-HSCs were either platelet or platelet-erythroid restricted (66%), compared to 10% of total HSCs (Carrelha et al., 2018). Gating using the identified markers therefore provides significantly improved PLT-HSC purification compared to previous markers, in addition to being transgene independent.

Marker-based quantification of HSC perturbation

Both aging and chronic inflammation lead to declining HSC function (Bogeska et al., 2022), which has led to the hypothesis that increased inflammation is a driver of HSC aging. A key characteristic of aged HSCs is their molecular and functional platelet or platelet-myeloid bias (Cho

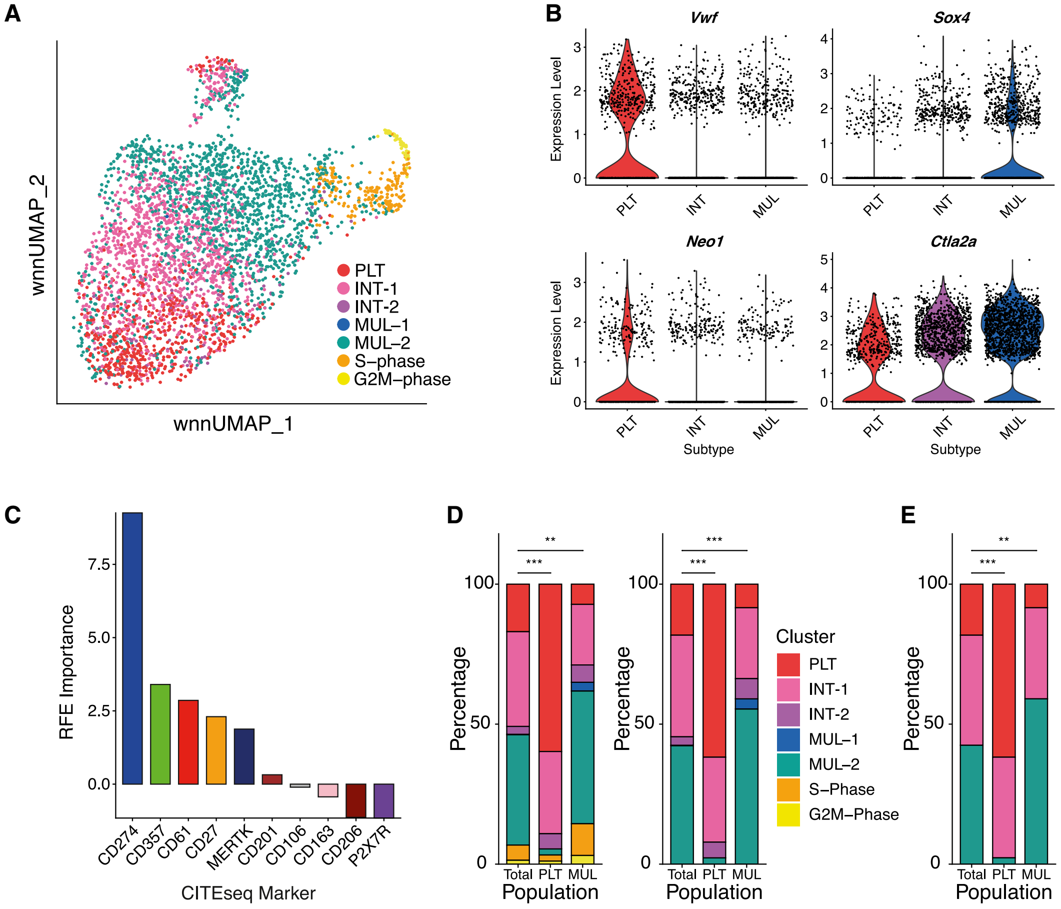

Figure 4. HSC subpopulation marker is conserved during aging

(A) Weighted nearest neighbor (WNN) UMAP projection of HSCs from 24-month-old mice. Cells were annotated by anchor-based transferring of 8-week-old mice HSC labels from Figure 2A.

(B) Expression levels of selected lineage potential signature genes in label-transferred clusters.

(C) Importance scores of CITE-seq markers for separating the PLT and MUL clusters, calculated using recursive feature elimination (RFE).

(D) *In silico* gating simulation of aged HSCs from (A) using the top four RFE-ranked markers from (B). The proportion of total, PLT- and MUL-gated cells belonging to each cluster from (A) is shown, including (left) and excluding (right) the cycling clusters.

(E) Cluster identities from (D) were grouped into PLT, INT (INT-1 and INT-2 combined), and MUL (MUL-1 and MUL-2 combined) populations.

Statistical comparisons performed using chi-squared tests for *in silico* gating; $^{**}p < 0.005$, $^{***}p < 0.001$.

et al., 2008; Grover et al., 2016). However, it is currently not clear if this aspect of the aging process is inflammation driven in the same manner as declining HSC function.

To address this question, we first tested if the identified surface markers could discriminate HSC subtypes in old mice. For this purpose, we profiled HSCs from 24-month-old mice using the same DOGMA-seq workflow and used anchor-based label transfer to identify aged HSCs molecularly similar to the subtypes defined in the young sample (Figures 4A and 4B). Using this annotation, we performed RFE analysis, observing that the same 4 markers were the top discriminators (Figure 4C). *In silico* gating (Figures 4D and 4E) showed similar enrichment of PLT- and MUL-HSCs using the 4 markers as in young mice (Figures 2F and 2G), supporting their robustness across age, the main difference being higher enrichment for PLT-HSCs in old mice, likely reflecting the higher abundance of this subtype.

To next test if the 4-marker panel could capture changes to HSC composition during normal aging, we used it to quantify PLT- and MUL-HSCs by flow cytometry, observing that the proportion of phenotypic PLT-HSCs was increased from 10% in young (2 months old) mice to 70% in 24-month-old mice (Figures 5A and 5B), in line with previous quantification by single-HSC transplantation (Grover et al., 2016). In contrast, using the previously characterized

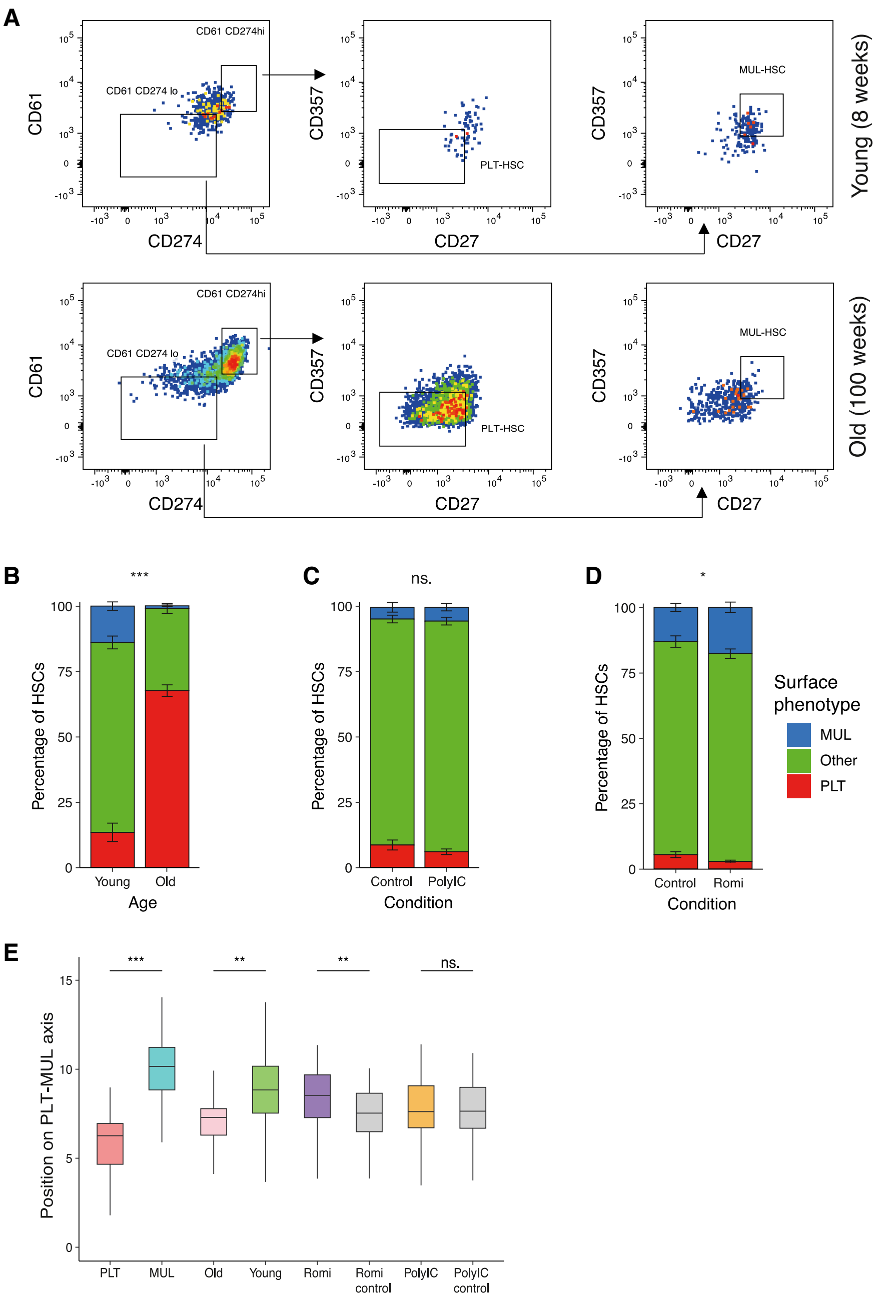

Figure 5. FACS and qPCR panel discriminate HSC subtypes

(A) HSC subtype flow cytometry of young (8 weeks) and aged (100 weeks) mouse HSCs.

(B) Proportion of gated PLT ($CD61^{hi}CD274^{hi}CD357^{lo}CD27^{lo}$), MUL ($CD61^{lo}CD274^{lo}CD357^{hi}CD27^{hi}$), and non-PLT/MUL-gated (other) HSCs in young and aged mice.

(C) Proportion of gated PLT- and MUL-HSCs as in (A) in poly(I-C)-treated and control mice.

(D) Proportion of gated PLT- and MUL-HSCs as in (A) in romiplostim-treated and control mice.

(E) Lineage bias scores of the FACS-sorted PLT- and MUL-HSCs (Figure 2C), young, aged, poly(I-C) treated, and control HSCs. Scores were calculated by projecting individual cells onto a Slingshot trajectory connecting the PLT- and MUL-HSC populations using principal components derived from microfluidics RT-qPCR gene expression values (Figure 3C).

Mouse number: young, $N = 5$; old, $N = 4$; romiplostim, $N = 4$; romiplostim control, $N = 4$; poly(I-C), $N = 8$; poly(I-C) control, $N = 4$. qPCR HSC number: MUL, $N = 62$; PLT, $N = 63$; old, $N = 80$; young, $N = 85$; romiplostim, $N = 89$; romiplostim control, $N = 89$; poly(I-C), $N = 240$; poly(I-C) control, $N = 263$. Statistical comparisons performed using multivariate analyses (MANOVA) for HSC subtype staining and unpaired two-tailed t tests for lineage bias scores; $^{*}p < 0.05$, $^{**}p < 0.005$, $^{***}p < 0.001$. Data are presented as mean ± standard deviation.

model of chronic inflammation based on prolonged treatment with poly(I-C), which leads to decline in HSC function (Bogeska et al., 2022), no significant changes to the proportion of HSC subtypes were observed (Figure 5C). Conversely, treatment with the thrombopoietin (TPO) agonist romiplostim (Romi) can enhance multi-lineage output from a depleted HSC pool in aplastic anemia (Lee et al., 2019) and in TPO-deficient mice (Nakamura-Ishizu et al., 2021). To test if Romi stimulation affects the phenotypic composition of the HSC compartment, we Romi treated adult mice. Across 4 weeks of semi-weekly Romi treatment, we observed increased platelet counts (Figure S6A), accompanied by expansion of the HSC and downstream progenitor compartments (Figure S6B). Our marker panel also revealed a significant increase in the proportion of MUL-HSCs relative to controls (Figure 5D).

To further substantiate these findings, we used microfluidics-based RT-qPCR, comparing purified MUL- and PLT-HSCs to aged and treated HSCs. To quantify any changes to lineage bias, we projected each single-cell population onto

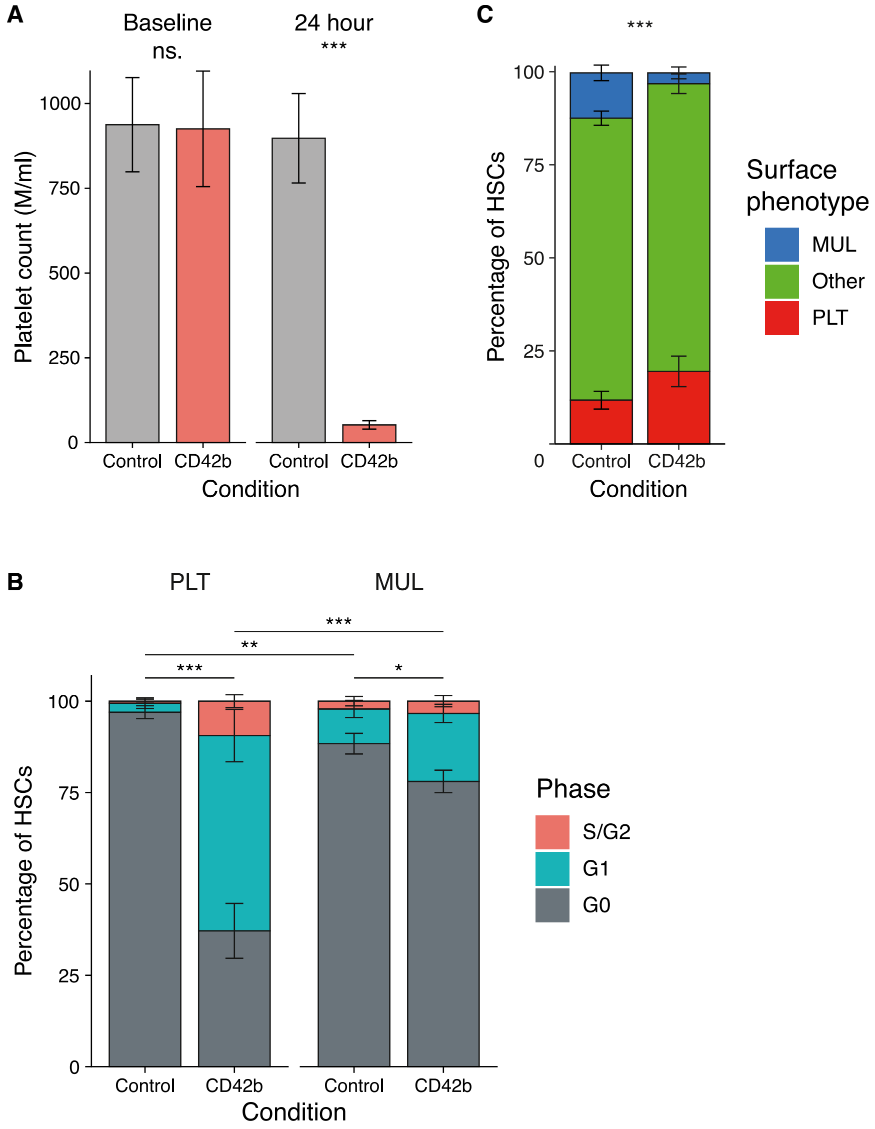

Figure 6. PLT-HSCs are activated by acute platelet depletion
(A) Peripheral blood platelet count following anti-CD42b treatment or control.
(B) Cell cycle phase distribution of PLT- and MUL-HSCs in CD42b antibody-treated and control mice.
(C) Proportion of gated PLT ($CD61^{hi}CD274^{hi}CD357^{lo}CD27^{lo}$), MUL ($CD61^{lo}CD274^{lo}CD357^{hi}CD27^{hi}$), and non-PLT/MUL-gated (other) HSCs after platelet depletion.
Mouse number: CD42b treated, $N = 6$; control: $N = 4$. Statistical comparisons performed using unpaired two-tailed t tests for platelet count, multivariate analyses (MANOVA) for cell cycle analysis and FACS gating, $*p < 0.05$, $**p < 0.005$, $***p < 0.001$. Data are presented as mean ± standard deviation.

the axis connecting the fluorescence-activated cell sorting (FACS)-purified MUL- and PLT-HSCs and compared the projected positions along this axis between conditions. This confirmed a quantitative shift toward platelet bias in aged compared to young HSCs and increased multi-lineage signature following Romi treatment, with no significant difference between poly(I-C)-treated and control HSCs (Figure 5E).

Finally, we previously observed that *Vwf*-EGFP+ HSCs were preferentially induced to proliferate compared to *Vwf*-EGFP− HSCs after anti-CD42b-mediated platelet depletion (Luis et al., 2023). While this would be consistent with selective activation of PLT-HSCs, these only comprise 30% of the *Vwf*-EGFP+ HSC population (as discussed, Figure 3H), making a definitive conclusion difficult. Using the 4-marker panel to analyze platelet-depleted mice (Figure 6A), we observed a significant induction of PLT-HSC cycling (Figure 6B), leading to increased prevalence of PLT-HSCs in platelet-depleted compared to control mice (Figure 6C). In contrast, MUL-HSCs were more proliferative at baseline but showed only a modest increase in cell cycling after platelet depletion.

DISCUSSION

Previously, a number of cell surface markers such as CD41 (Gekas and Graf, 2013), CD150 (Morita et al., 2010), Neogenin-1 (Gulati et al., 2019), and CD62P (Ross et al., 2024) have been associated with PLT- or platelet-myeloid-biased HSCs at the population level, but the extent to which

these enrich PLT-HSCs at the single-cell level remains unclear. Currently, the best characterized strategy for PLT-HSC purification involves using a *Vwf* reporter. This partitions essentially all PLT-HSCs into the *Vwf*+ fraction (Carrelha et al., 2018), which is sufficient to generate platelet bias at the population level (Sanjuan-Pla et al., 2013). However, by single-cell transplantation, only 30% of *Vwf*+ HSCs are platelet biased, with the remaining 70% showing multi-lineage, myeloid-biased, and lymphoid-biased reconstitution patterns (Carrelha et al., 2018). We here used multiome profiling to identify a combination of cell surface markers that can identify PLT-HSCs within the HSC population with improved accuracy, allowing PLT-HSC to be prospectively identified and purified to ca. 85% purity, independently of any transgene, allowing more accurate phenotyping of HSC subtypes across multiple scenarios, including genetic and metabolic perturbations, aging and inflammation, as well as more accurate identification of cells-of-origin of hematological malignancies.

Hematopoietic stem cell aging is a complex process that involves both shifts in the abundance of HSC subtypes and changes to HSC function, and inflammation has been proposed to drive this process. However, comparing the effects of aging and chronic inflammation on HSC heterogeneity, we did not find that chronic inflammation increased HSC platelet bias using either cell surface markers or gene expression analysis. This suggests that while important for aging, an interferon-driven, pro-inflammatory response is not in itself sufficient to drive the age-associated change to HSC composition, which may involve a combination of extrinsic drivers such as IL-1 (Caiado and Manz, 2024) or TGF-β (Valletta et al., 2020), as well as changes to the bone marrow stromal microenvironment. While it is important to emphasize that a single inflammatory signal does not mimic the aging, this indicates that loss of HSC functionality, while observed in aging, does not necessarily derive from inflammation alone but is generated through the integrated action of multiple age-associated processes.

Platelet depletion by anti-CD42b antibody treatment releases IL-1, leading to niche-mediated HSC proliferation (Luis et al., 2023). We here find that in this scenario, PLT-HSCs are preferentially activated, consistent with the previous observation that *Vwf*+ HSCs are efficiently induced to expand, but providing greater specificity. In contrast, Romi treatment, which acts through the TPO receptor Mpl, induced a significant shift of the HSC compartment toward a multi-lineage phenotype, as measured using both cell surface and molecular markers. This indicates that sustained high-level Mpl activation can provide a selective advantage to MUL-HSCs, revealing a potential route to rebalancing the aging HSC compartment. While further work will be needed to explore this possibility, and in particular to understand if Romi selectively expands MUL-HSCs or drives PLT-HSCs toward differentiation, these results, along with those discussed earlier, show that an improved molecular phenotyping framework for dissecting HSC lineage bias can guide future modeling and perturbation studies, with an important next step being to test the identified markers on human cells, and if needed identify additional ones, in order to develop a similar panel for human PLT- and MUL-HSCs, as recently identified (Aksoz et al., 2024).

RESOURCE AVAILABILITY

Lead contact

Further information and requests for resources and reagents should be directed to and will be fulfilled by the lead contact, Claus Nerlov.

Materials availability

This study did not generate new unique reagents.

Data and code availability

Code for Fluidigm qPCR data analysis is available from GitHub repository NerlovLab/Fluidigm_PCA_projection. The sequencing data generated in this study have been deposited in the Gene Expression Omnibus (GEO) under accession numbers GEO: GSE306075 and GEO: GSE328367. Raw microfluidics-based RT-qPCR data are available from GitHub: NerlovLab/Fluidigm_PCA_projection.

ACKNOWLEDGMENTS

We thank Xinran Huang and Yifan Bao from the MRC WIMM and Fenia Fotopoulou, Rebecca Lipp, Ian Ghezzi, Sena Alptekin, and Lena Bognar for assistance with poly(I-C) injections, cell sorting, and bone marrow harvests. We also acknowledge the Oxford Biomedical Services (BMS) for support with animal work and the WIMM Flow Cytometry Facility for expert assistance with cell sorting. This work was supported by MRC Unit grants (MC_UU_12009/7 and MC_UU_00029/9) to C.N.

AUTHOR CONTRIBUTIONS

B.Z. conceived and designed the study and performed the majority of experiments, bioinformatic analyses, and data interpretations. Y.M. performed the HSC clonal expansion and bulk sequencing experiments, maintained the *Vwf*-EGFP mouse colony, contributed to bone marrow harvests, and provided daily supervision. E.R.C. performed poly(I-C) injections and contributed to bone marrow harvests. X.R. contributed to a substantial number of bone marrow harvests. A.F. maintained the *Gata1*-EGFP mouse colony. M.M. led the poly(I-C) experiments as co-PI. C.N. conceived and designed the study and supervised the projects as principal investigator.

DECLARATION OF INTERESTS

The authors declare no competing interests.

STAR★METHODS

Detailed methods are provided in the online version of this paper and include the following:

- KEY RESOURCES TABLE
- EXPERIMENTAL MODEL AND STUDY PARTICIPANT DETAILS
  - Animals
- METHOD DETAILS
  - Flow cytometry
  - Bone marrow preparation
  - Cell cycle analysis
  - Single-cell transplantation
  - Peripheral blood cell preparation for FACS
  - CITEseq antibody conjugation
  - DOGMAseq
  - Fluidigm single-cell qRT-PCR
  - Poly(I-C) treatment
  - Romiplostim treatment
  - Anti-CD42b antibody platelet depletion
  - Candidate CITEseq and Fluidigm marker identification
  - Single-cell multiome data processing
  - WNN clustering and dimensional reduction
  - In silico HSC subtype scoring
  - In silico marker selection and gating
  - Multiome data label transfer
- QUANTIFICATION AND STATISTICAL ANALYSIS
  - Multiome data analysis
  - Figure-level statistical analyses

SUPPLEMENTAL INFORMATION

Supplemental information can be found online at https://doi.org/10.1016/j.stemcr.2026.102959.

Received: December 4, 2025
Revised: May 13, 2026
Accepted: May 14, 2026
Published: June 11, 2026

REFERENCES

Aksöz, M., Gafencu, G.A., Stoilova, B., Buono, M., Zhang, Y., Turkalj, S., Meng, Y., Jakobsen, N.A., Metzner, M., Clark, S.A., et al. (2024). Hematopoietic stem cell heterogeneity and age-associated platelet bias are evolutionarily conserved. Sci. Immunol. *9*, eadk3469.

Amemiya, H.M., Kundaje, A., and Boyle, A.P. (2019). The ENCODE Blacklist: Identification of Problematic Regions of the Genome. Sci. Rep. *9*, 9354.

Bogeska, R., Mikecin, A.M., Kaschutnig, P., Fawaz, M., Büchler-Schäff, M., Le, D., Ganuza, M., Vollmer, A., Paffenholz, S.V., Asada, N., et al. (2022). Inflammatory exposure drives long-lived impairment of hematopoietic stem cell self-renewal activity and accelerated aging. Cell Stem Cell *29*, 1273–1284.e8.

Caiado, F., and Manz, M.G. (2024). IL-1 in aging and pathologies of hematopoietic stem cells. Blood *144*, 368–377.

Carrelha, J., Meng, Y., Kettyle, L.M., Luis, T.C., Norfo, R., Alcolea, V., Boukarabila, H., Grasso, F., Gambardella, A., Grover, A., et al. (2018). Hierarchically related lineage-restricted fates of multipotent haematopoietic stem cells. Nature *554*, 106–111.

Cho, R.H., Sieburg, H.B., and Muller-Sieburg, C.E. (2008). A new mechanism for the aging of hematopoietic stem cells: aging changes the clonal composition of the stem cell compartment but not individual stem cells. Blood *111*, 5553–5561.

Drissen, R., Buza-Vidas, N., Woll, P., Thongjuea, S., Gambardella, A., Giustacchini, A., Mancini, E., Zriwil, A., Lutteropp, M., Grover, A., et al. (2016). Distinct myeloid progenitor-differentiation pathways identified through single-cell RNA sequencing. Nat. Immunol. *17*, 666–676.

Finak, G., McDavid, A., Yajima, M., Deng, J., Gersuk, V., Shalek, A.K., Slichter, C.K., Miller, H.W., McElrath, M.J., Prlic, M., Linsley, P.S., and Gottardo, R. (2015). MAST: a flexible statistical framework for assessing transcriptional changes and characterizing heterogeneity in single-cell RNA sequencing data. Genome Biol *16*, 278.

Gekas, C., and Graf, T. (2013). CD41 expression marks myeloid-biased adult hematopoietic stem cells and increases with age. Blood *121*, 4463–4472.

Grieshaber-Bouyer, R., Radtke, F.A., Cunin, P., Stifano, G., Levescot, A., Vijaykumar, B., Nelson-Maney, N., Blaustein, R.B., Monach, P.A., and Nigrovic, P.A.; ImmGen Consortium (2021). The neutrotime transcriptional signature defines a single continuum of neutrophils across biological compartments. Nat. Commun. *12*, 2856.

Grover, A., Sanjuan-Pla, A., Thongjuea, S., Carrelha, J., Giustacchini, A., Gambardella, A., Macaulay, I., Mancini, E., Luis, T.C., Mead, A., et al. (2016). Single-cell RNA sequencing reveals molecular and functional platelet bias of aged haematopoietic stem cells. Nat. Commun. *7*, 11075.

Gulati, G.S., Zukowska, M., Noh, J.J., Zhang, A., Wesche, D.J., Sinha, R., George, B.M., Weissman, I.L., and Szade, K. (2019). Neogenin-1 distinguishes between myeloid-biased and balanced Hoxb5(+) mouse long-term hematopoietic stem cells. Proc. Natl. Acad. Sci. USA *116*, 25115–25125.

Hao, Y., Hao, S., Andersen-Nissen, E., Mauck, W.M., 3rd, Zheng, S., Butler, A., Lee, M.J., Wilk, A.J., Darby, C., Zager, M., et al. (2021). Integrated analysis of multimodal single-cell data. Cell *184*, 3573–3587.e29.

Hao, Y., Stuart, T., Kowalski, M.H., Choudhary, S., Hoffman, P., Hartman, A., Srivastava, A., Molla, G., Madad, S., Fernandez-Granda, C., and Satija, R. (2024). Dictionary learning for integrative, multimodal and scalable single-cell analysis. Nat. Biotechnol. *42*, 293–304.

Kimmerlin, Q., Usart, M., Fonseca, T., Hao-Shen, H., and Skoda, R.C. (2024). JAK2-V617F-Driven Myeloproliferative Neoplasms (MPN) Are Initiated By a Subset of HSCs Marked By High Expression of the Vwf-GFP Reporter Gene. Blood *144*, 751.

Lee, J.W., Lee, S.E., Jung, C.W., Park, S., Keta, H., Park, S.K., Kim, J.A., Oh, I.H., and Jang, J.H. (2019). Romiplostim in patients with refractory aplastic anaemia previously treated with immunosuppressive therapy: a dose-finding and long-term treatment phase 2 trial. Lancet Haematol. *6*, e562–e572.

Love, M.I., Huber, W., and Anders, S. (2014). Moderated estimation of fold change and dispersion for RNA-seq data with DESeq2. Genome Biol. *15*, 550.

Luis, T.C., Barkas, N., Carrelha, J., Giustacchini, A., Mazzi, S., Norfo, R., Wu, B., Aliouat, A., Guerrero, J.A., Rodriguez-Meira, A., et al. (2023). Perivascular niche cells sense thrombocytopenia and activate hematopoietic stem cells in an IL-1 dependent manner. Nat. Commun. *14*, 6062.

Meng, Y., Carrelha, J., Drissen, R., Ren, X., Zhang, B., Gambardella, A., Valletta, S., Thongjuea, S., Jacobsen, S.E., and Nerlov, C. (2023). Epigenetic programming defines haematopoietic stem cell fate restriction. Nat. Cell Biol. *25*, 812–822.

Meng, Y., and Nerlov, C. (2025). Epigenetic regulation of hematopoietic stem cell fate. Trends Cell Biol. *35*, 217–229.

Mimitou, E.P., Lareau, C.A., Chen, K.Y., Zorzetto-Fernandes, A.L., Hao, Y., Takeshima, Y., Luo, W., Huang, T.S., Yeung, B.Z., Papalexi, E., et al. (2021). Scalable, multimodal profiling of chromatin accessibility, gene expression and protein levels in single cells. Nat. Biotechnol. *39*, 1246–1258.

Morita, Y., Ema, H., and Nakauchi, H. (2010). Heterogeneity and hierarchy within the most primitive hematopoietic stem cell compartment. J. Exp. Med. *207*, 1173–1182.

Nakamura-Ishizu, A., Chin, D.W.L., Matsumura, T., Tan, D.Q., Mochizuki-Kashio, M., Jianwen, D., and Suda, T. (2021). Prolonged maintenance of hematopoietic stem cells that escape from thrombopoietin deprivation. Blood *137*, 2609–2620.

Pei, W., Shang, F., Wang, X., Fanti, A.K., Greco, A., Busch, K., Klapproth, K., Zhang, Q., Quedenau, C., Sauer, S., et al. (2020). Resolving Fates and Single-Cell Transcriptomes of Hematopoietic Stem Cell Clones by PolyloxExpress Barcoding. Cell Stem Cell *27*, 383–395.e8.

Pinho, S., Marchand, T., Yang, E., Wei, Q., Nerlov, C., and Frenette, P.S. (2018). Lineage-Biased Hematopoietic Stem Cells Are Regulated by Distinct Niches. Dev. Cell *44*, 634–641.e4.

Rodriguez-Fraticelli, A.E., Weinreb, C., Wang, S.W., Migueles, R.P., Jankovic, M., Usart, M., Klein, A.M., Lowell, S., and Camargo, F.D. (2020). Single-cell lineage tracing unveils a role for TCF15 in haematopoiesis. Nature *583*, 585–589.

Ross, J.B., Myers, L.M., Noh, J.J., Collins, M.M., Carmody, A.B., Messer, R.J., Dhuey, E., Hasenkrug, K.J., and Weissman, I.L. (2024). Depleting myeloid-biased haematopoietic stem cells rejuvenates aged immunity. Nature *628*, 162–170.

Sanjuan-Pla, A., Macaulay, I.C., Jensen, C.T., Woll, P.S., Luis, T.C., Mead, A., Moore, S., Carella, C., Matsuoka, S., Bouriez Jones, T., et al. (2013). Platelet-biased stem cells reside at the apex of the haematopoietic stem-cell hierarchy. Nature *502*, 232–236.

Schep, A.N., Wu, B., Buenrostro, J.D., and Greenleaf, W.J. (2017). chromVAR: inferring transcription-factor-associated accessibility from single-cell epigenomic data. Nat. Methods *14*, 975–978.

Tirosh, I., Venteicher, A.S., Hebert, C., Escalante, L.E., Patel, A.P., Yizhak, K., Fisher, J.M., Rodman, C., Mount, C., Filbin, M.G., et al. (2016). Single-cell RNA-seq supports a developmental hierarchy in human oligodendroglioma. Nature *539*, 309–313.

Valletta, S., Thomas, A., Meng, Y., Ren, X., Drissen, R., Sengül, H., Di Genua, C., and Nerlov, C. (2020). Micro-environmental sensing by bone marrow stroma identifies IL-6 and TGF beta 1 as regulators of hematopoietic ageing. Nat. Commun. *11*, 4075.

Yu, G., Wang, L.G., Han, Y., and He, Q.Y. (2012). clusterProfiler: an R package for comparing biological themes among gene clusters. OMICS *16*, 284–287.

STAR★METHODS

KEY RESOURCES TABLE

REAGENT or RESOURCE	SOURCE	IDENTIFIER
**Antibodies**		
Anti-CD27 antibody-derived tag (ADT) reagent	BioLegend	Cat#124235; RRID: AB_2750344
Anti-CD61 antibody-derived tag (ADT) reagent	BioLegend	Cat#104327; RRID: AB_2832324
Anti-CD68 antibody-derived tag (ADT) reagent	BioLegend	Cat#137001; RRID: AB_2044003
Anti-CD106 antibody-derived tag (ADT) reagent	BioLegend	Cat#105701; RRID: AB_313202
Anti-CD163 antibody-derived tag (ADT) reagent	BioLegend	Cat#156702; RRID: AB_2800711
Anti-CD183 antibody-derived tag (ADT) reagent	BioLegend	Cat#155902; RRID: AB_2750166
Anti-CD201 antibody-derived tag (ADT) reagent	BioLegend	Cat#141509; RRID: AB_2800655
Anti-CD206 antibody-derived tag (ADT) reagent	BioLegend	Cat#141705; RRID: AB_10896421
Anti-CD274 antibody-derived tag (ADT) reagent	BioLegend	Cat#153604; RRID: AB_2783125
Anti-CD357 antibody-derived tag (ADT) reagent	BioLegend	Cat#126319; RRID: AB_2734195
Anti-P2X7R antibody-derived tag (ADT) reagent	BioLegend	Cat#148711; RRID: AB_2800683
Anti-MERTK antibody-derived tag (ADT) reagent	BioLegend	Cat#151502; RRID: AB_2566624
Anti-B220 antibody, PECy5 (lineage cocktail)	BioLegend	Cat#103210; RRID: AB_312995
Anti-CD4 antibody, PECy5 (lineage cocktail)	eBioscience	Cat#15-0042-82; RRID: AB_468698
Anti-CD8 antibody, PECy5 (lineage cocktail)	eBioscience	Cat#15-0081-82; RRID: AB_468706
Anti-Mac-1 antibody, PECy5 (lineage cocktail)	eBioscience	Cat#15-0112-82; RRID: AB_468714
Anti-Gr-1 antibody, PECy5 (lineage cocktail)	eBioscience	Cat#15-5931-82; RRID: AB_468813
Anti-Ter119 antibody, PECy5 (lineage cocktail)	eBioscience	Cat#15-5921-82; RRID: AB_468810
Anti-CD5 antibody, PECy5 (lineage cocktail)	BioLegend	Cat#100610; RRID: AB_312739
Anti-*c*-Kit antibody, APCe780 (Bone marrow)	eBioscience	Cat#47-1171-82; RRID: AB_1272177
Anti-CD150 antibody, BV785 (Bone marrow)	BioLegend	Cat#115937; RRID: AB_2565962
Anti-CD34 antibody, AF700 (Bone marrow)	eBioscience	Cat#56-0341-82; RRID: AB_493998
Anti-CD45.1 antibody, BV650 (Bone marrow)	BioLegend	Cat#110736; RRID: AB_2562564
Anti-CD45.2 antibody, AF700 (Bone marrow)	eBioscience	Cat#56-0454-82; RRID: AB_657752
Anti-Sca-1 antibody, BV421 (Bone marrow)	BioLegend	Cat#108128; RRID: AB_2563064
Anti-CD48 antibody, BV510 (Bone marrow)	BioLegend	Cat#103443; RRID: AB_2650826
Anti-CD274 antibody, BV605 (Bone marrow)	BioLegend	Cat#153606; RRID: AB_2814056
Anti-CD61 antibody, PE (Bone marrow)	BioLegend	Cat#104307; RRID: AB_313084
Anti-CD357 antibody, PECy7 (Bone marrow)	BioLegend	Cat#126318; RRID: AB_2563386
Anti-CD27 antibody, APC (Bone marrow)	BioLegend	Cat#124212; RRID: AB_2073425
Anti-Ki-67 antibody, BV650 (Bone marrow)	BD Biosciences	Cat#563757; RRID: AB_2688008

(*Continued on next page*)

Continued

REAGENT or RESOURCE	SOURCE	IDENTIFIER
Anti-Mac1 antibody, PECy7 (WBC)	eBioscience	Cat#25-0112-81; RRID: AB_469587
Anti-CD19 antibody, eF450 (WBC)	eBioscience	Cat#48-0193-80; RRID: AB_2637304
Anti-CD4 antibody, APC (WBC)	eBioscience	Cat#17-0042-82; RRID: AB_469323
Anti-CD8a antibody, APCe780 (WBC)	eBioscience	Cat#47-0081-82; RRID: AB_1272185
Anti-NK1.1 antibody, PECy5 (WBC)	BioLegend	Cat#108716; RRID: AB_493590
Anti-CD41 antibody, BV421 (Platelet/RBC)	BioLegend	Cat#133911; RRID: AB_10960744
Anti-Ter119 antibody, APC (Platelet/RBC)	eBioscience	Cat#17-5921-82; RRID: AB_469473
Anti-CD42b antibody for platelet depletion	Emfret Analytics	Cat#R300
Non-immune rat IgG control antibody	Emfret Analytics	Cat#C301
**Chemicals, peptides, and recombinant proteins**		
CD117 magnetic beads	Miltenyi	Cat#130-097-146
MACS LS column	Miltenyi	Cat#130-042-401
FcR blocking agent	Miltenyi	Cat#130-092-575
Cytofix/Cytoperm buffer	BD	Cat#554714
Single-colour positive and negative compensation beads	BD	Cat#51-90-9001189; Cat#51-90-9001291
UltraComp eBeads	ThermoFisher	Cat#U20250
RNase inhibitor	ThermoFisher	Cat#PN-E00382
1× nuclei buffer	10× Genomics	Cat#1000285
KAPA HiFi Mix	Roche	Cat#KK2601
SPRI beads	Beckman Coulter	Cat#B23318
TapeStation D5000 screening tapes	Agilent	Cat#5067-5588
NEBNext library quant kit	NEB	Cat#E7630
Amicon Ultra 50 kDa cut-off columns	Millipore	Cat#UFC505024
SYBR Safe dye	Invitrogen	Cat#S33102
Dextran	Sigma	Cat#31392
Ammonium chloride	Stemcell Technologies	Cat#07850
High molecular weight poly(I-C)	As described (Bogeska et al., 2022)	N/A
Romiplostim (Nplate)	N/A	N/A
**Critical commercial assays**		
Lightning-Link kit	Abcam	Cat#ab102921
Fluidigm 192.24 IFC platform	StandardBioTools	Cat#100-6170
10× Multiome pipeline	10× Genomics	CG000338
**Deposited data**		
Sequencing data generated in this study	This paper	GEO: GSE306075; GEO: GSE328367

(*Continued on next page*)

Continued

REAGENT or RESOURCE	SOURCE	IDENTIFIER
Raw microfluidics-based qRT-PCR data	This paper	GitHub repository NerlovLab/ Fluidigm_PCA_projection
**Experimental models: Organisms/strains**		
Mouse: C57BL/6J wild type	Charles River UK	Model 632; JAX stock#000664; RRID: IMSR_JAX:000664
Mouse: *Vwf*-EGFP reporter mice; Tg(*Vwf*-EGFP)2Nerl	Sten Eirik W. Jacobsen; Sanjuan-Pla et al. (2013)	MGI:6505537
Mouse: *Gata1*-EGFP mice, CD45.1 allotype	Claus Nerlov; Drissen et al. (2016)	N/A
**Oligonucleotides**		
Pre-amp	IDT	CCTTGGCACCCGAGAATTCC
SI-PCR	IDT	AATGATACGGCGACCACCGAGATCTAC ACTCTTTCCCTACACGACGCTC
RPxx (sample index)	IDT	CAAGCAGAAGACGGCATACGAGAT/ SampleIndex/GTGACTGGAGTTCCT TGGCACCC GAGAATTCCA
ADT	IDT	/5Biosg/CCTTGGCACCGAGAATCCA/ Barcode/GAAAAAAAAAAAAAAAAA AAAAAAAAAAAA
TaqMan PCR probes	Thermo Fisher Scientific	See Table S5
**Software and algorithms**		
Cellranger-arc	10×	N/A
CITE-seq-Count	Grieshaber-Bouyer et al. (2021)	N/A
Seurat v5	Hao et al. (2024)	N/A
ChromVAR	Schep et al. (2017)	N/A
Signac	N/A	N/A
DESeq2	N/A	N/A
MAST	Finak et al. (2015)	N/A
clusterProfiler	Yu et al. (2012)	N/A
Slingshot	N/A	N/A
pheatmap	R package	N/A
Fluidigm qPCR data analysis code	This paper	GitHub repository NerlovLab/ Fluidigm_PCA_projection
**Other**		
BD Fortessa	BD	N/A
BD Aria III, Aria Fusion, and S6 cell sorters	BD	N/A
Sony ID7000 spectral analyser	Sony	N/A
Horiba Pentra Haematology Analyser ES60	Horiba	N/A

EXPERIMENTAL MODEL AND STUDY PARTICIPANT DETAILS

Animals

All animal procedures, including breeding, maintenance, blood sampling and culling were performed, in accordance with UK Home Office regulations and the U.K. Animals (Scientific Procedures) Act, 1986, and with ethical approval by the University of Oxford Medical Sciences Division Animal Welfare and Ethical Review Body, under UK Home Office project license PP2240412. Experimental mice were all from the C57BL6/J strain. For candidate FACS antibody validation, 8-week *Vwf*-EGFP mice (Sanjuan-Pla et al., 2013) were used. For transplantations, *Gata1*-EGFP mice (Drissen et al., 2016) with a CD45.1 allotype aged 8 to 12 weeks were used as HSC donors. Wild type CD45.2 recipient mice, aged 8 weeks, were obtained from Charles River UK (Model 632). These wild type C57Bl6/J mice were also used for single-cell sequencing, Fluidigm single-cell qPCR, Poly(I-C) and Romiplostim treatments.

METHOD DETAILS

Flow cytometry

Flow cytometric analysis was performed using BD Fortessa. Single-colour positive and negative compensation beads (BD, 51-90-9001189 & 51-90-9001291) were stained with 1:400 antibodies and used for creating the FACS panels. Fresh fluorescence-minus-one (FMO) controls were included in each individual experiment to guide gating and compensation. HSC sorting was carried out using BD Aria III, Aria Fusion, and S6 cell sorters. Bulk purity mode was used for sorting samples destined for bulk sequencing, DOGMAseq and bulk transplantation, while single-cell purity mode with indexing was employed for single-cell transplantation and single-cell qPCR experiments.

Spectral flow cytometry was performed using a Sony ID7000 spectral analyser. Single-colour reference controls were prepared using UltraComp eBeads (ThermoFisher, U20250) stained with individual antibodies and used for spectral unmixing and panel setup. For permeabilised panels, single-colour controls were subjected to the same fixation and permeabilization procedures as experimental samples. Spectral data were unmixed using the instrument's unmixing algorithm with reference controls acquired under identical settings. Both freshly isolated and permeabilised bone marrow samples treated with anti-CD42b antibody were analyzed by spectral flow cytometry.

Bone marrow preparation

Bone marrow was harvested from sternum, femur, tibia and pelvis. Bones were crushed in FACS media (PBS with 5% FCS and 2 mM EDTA), and filtered through a 50 μm sterile filter. Sample leukocyte concentrations were measured by a Horiba Pentra Haematology Analyser ES60. Whole bone marrow (BM) cells were c-Kit enriched using MACS magnetic separation. The filtered BM cells were pelleted by centrifugation at 500*g* for 5 min and resuspended at a concentration of 1 million (M) WBCs/μL of FACS media. The cells were then mixed with CD117 magnetic beads (Miltenyi, 130-097-146) at a ratio of 2.5 μL/100 M WBCs and incubated on ice for 20 min. Subsequently, cells were washed, resuspended in 4 mL of FACS media, and passed through a MACS LS column (Miltenyi, 130-042-401). The column was washed three times with 3 mL of FACS media, removed from the magnet, and the c-Kit enriched cells were collected by flushing 8 mL of FACS media through the column into a new tube. The c-Kit enriched cells were first incubated with 1× FcR blocking agent (Miltenyi, 130-092-575) for 10 min and mixed with a 2× antibody (FACS + CITE-seq) cocktail, incubated further for 25 min, washed, and resuspended in FACS media containing 0.525 μg/μL 7-AAD for FACS, or proceeded for cell cycle analysis.

Cell cycle analysis

Stained bone marrow cells were permeabilised by Cytofix/Cytoperm buffer (BD, 554714) on ice for 20 min. Permeabilised cells were then washed in 1× Perm/Wash buffer (BD, 554714) twice, resuspended, and stained with Ki-67 antibody overnight at 4°C. The next day, cells were stained with 1 μg/mL DAPI on ice for 10 min and resuspended in wash buffer for FACS.

Single-cell transplantation

Single HSCs were sorted into 100μL of injection media (IMDM Pen/Strep L-Glutamine 0.1mM 2-Mercaptoethanol 20% BIT9500) in a U-bottom 96-well tissue culture plate. After sorting, an additional 100 μL of injection media containing 200,000 fresh CD45.2 competitor BM cells were added to each well, bringing the total injection volume to 200μL. The cells were then loaded into a 30G needle (Omnican50, B.Braun 9151125S) and injected intravenously into lethally irra-

diated (10.5Gray) recipients via the tail vein. The recipients were then given Baytril water for 4 weeks to prevent infection. PB was collected from the tail vein at week 6, 10, 16 for lineage reconstitution analysis (Platelet, Erythrocyte, Myeloid cell, T4 cell, T8 cell and B cell). PB lineage reconstitution was defined as > 0.1% donor chimerism within a given lineage. HSC fate restriction was defined by long-term reconstitution of peripheral blood cell types, as described (Carrelha et al., 2018).

Peripheral blood cell preparation for FACS

Peripheral blood (PB) was collected from the tail vein into heparin-coated tubes. To collect platelets and erythrocytes, blood was centrifuged for 10 min at 100 g; all the supernatant and 1 μL erythrocytes were transferred into a new tube containing 25 μL PB media (PBS 1% FCS 2 mM EDTA). To collect WBCs, the remaining blood was gently mixed with 100 μL PB media and 200 μL 2% Dextran (Sigma, 31392) and incubated at 37°C for 40 min to pellet red blood cells (RBCs). The supernatant was then transferred to a new tube, washed with 1 mL PB media and centrifuged at 1000 g. Next, the pellet was resuspended with 200 μL 0.8% ammonium chloride (Stemcell Technologies, 07850) for 4 min to lyse the remaining RBCs. Finally, the remaining WBCs were washed again with 1 mL PB media and resuspended in 25 μL PB media containing 1× FcR blocking reagents for 10 min before staining. Platelet, erythrocyte and WBC samples were stained with respective 2× antibody cocktail for 25 min, washed with 1 mL PB media and resuspended in PB media for FACS analysis.

CITEseq antibody conjugation

For candidates without off-the-shelf CITE-seq antibodies, a biotin-streptavidin conjugation was performed using the Lightning-Link kit (Abcam, ab102921). Antibody-derived tag (ADT) oligos (Table S3) were synthesised with a biotinylated 5′ tail (IDT) and reconstituted to 100 μM in TE buffer. To link the unconjugated antibody with streptavidin, 30 μL of modifier solution was added to 300 μL of 0.5 μg/μL antibody. The antibody was then mixed with 100 μg of lyophilised streptavidin and incubated overnight in the dark at room temperature (RT). The reaction was quenched by adding 30 μL of quenching solution followed by 40 μL of 0.1% Tween 20. To label each antibody molecule with 8 ADT oligos (as 4 docking sites per streptavidin molecule and 2 streptavidin per antibody), 150 μg of antibody was mixed with 8 nM biotinylated oligo and incubated overnight in the dark at RT. Conjugated antibodies were purified using Amicon Ultra 50 kDa cutoff columns (Millipore, UFC505024) according to the manufacturer's protocol. The purified antibody was recovered with 150 μL PBS and stored at 4°C. To verify the conjugation, 1 μg of tagged conjugated antibody was electrophoresed alongside 1 μL stock oligo, 1 μg unconjugated antibody and 1 μg conjugated antibody stored for 4 weeks at 4°C plus a sample thermally incubated at 37°C for 1 h, on a 4% agarose gel with SYBR Safe dye (Invitrogen, S33102) at 100 V for 60 min.

DOGMAseq

The workflow was adopted from (Mimitou et al., 2021) with modifications improving recovery rate for HSC samples. Cells were sorted into 200 μL of FACS media containing 0.2 U/μL RNase inhibitor (ThermoFisher, PN-EO0382) and centrifuged for 7 min at 500 g. The supernatant was carefully removed until 5 μL remained, then 45 μL of DIG permeabilisation buffer (Water, 20 mM Tris-HCl, 150 mM NaCl, 3 mM MgCl2, 0.01% Digitonin, 2 U/μL RNase inhibitor) was added to the pellet, mixed by pipetting three times, and incubated on ice for 5 min. The permeabilised cells (nuclei) were washed with 100 μL of DIG wash buffer (Water, 20 mM Tris-HCl, 150 mM NaCl, 3 mM MgCl2, 2 U/μL RNase inhibitor) and centrifuged for 7 min at 700 g. The supernatant was carefully aspirated until 2 μL remained, and 3 μL of 1× nuclei buffer (10× Genomics, 1000285) was added to resuspended the pellet. The samples were then processed through the 10× Multiome pipeline (CG000338) with modifications to generate the CITEseq library: At step 4.1 pre-amplification, 1 μL of a 0.2 μM pre-amp primer (Table S4) was added. To construct the CITEseq library, 30 μL of the pre-amplified library was mixed with 50 μL of 2× KAPA HiFi Mix (Roche, KK2601), 2.5 μL each of 10 μM SI-PCR and RPxx primers (Table S4), and topped up to 100 μL with water for the sample index PCR: 1: 95°C 3 min, 2: 95°C 20 s, 3: 60°C 30 s, 4: 72°C 20 s, 5: 72°C 5 min. Repeat 3–5 19 times. 6: 4°C hold. The PCR product was purified using SPRI beads (Beckman Coulter, B23318) at a ratio of 1.6×. The library qualities were analyzed by TapeStation D5000 screening tapes (Agilent, 5067–5588) and their concentrations were determined using the NEBNext library quant kit (NEB, E7630) with correction to their average fragment size between 100 and 1000 bp. For sequencing, ADT libraries were mixed with gene expression libraries at a 1:9 ratio based on molecular concentration. The sequencing parameters have followed the 10× Multiome guideline: 50-8-24-49, 25,000 paired reads per cell for ATAC libraries and 28-10-10-90, 20,000 paired reads per cell for RNA+CITEseq libraries.

Fluidigm single-cell qRT-PCR

Single-cell qRT-PCR was performed using the Fluidigm 192.24 IFC platform (StandardBioTools, 100–6170). First, single HSCs were FACS-sorted into 96-well PCR plates, each well containing 5 μL Fluidigm reverse transcription buffer (1×

Cell Direct 2× Reaction Mix, 12% SuperScript III RT/Platinum Taq Mix, 0.2 U/μL SUPERase·In RNase Inhibitor (Ambion, AM2694), 12% Buffer TE, 0.2× Taqman Assay mix). The plate was then incubated for the reverse transcription and targeted amplification by TaqMan PCR probes (Table S5): 1: 50°C 15 min: 2: 95°C 2 min, 3: 95°C 15 s, 4: 60°C 4 min. Repeat 3–4 22 times. 5: 4°C hold. After the incubation, 20 μL TE buffer was added to each well. Samples were then processed on a 192.24 IFC chip according to the manufacturer's protocol. Upon completion, a Ct-by-cell matrix was generated. The gene expression levels were normalised according to Fluidigm's official guide: an assumed baseline Ct value for negative results was set to Ct = 28; and any Ct > 28 were overwritten to 28. Then, 28 was subtracted from the Ct values and the delta Ct against the average Ct value of the two housekeeping genes was calculated. A clustered heatmap was generated using the R package pheatmap. To integrate data, delta Ct values were scaled and centered for each marker within each Fluidigm chip run before merging (Table S6). After integrating data from multiple chips, a PCA analysis was performed. A single-lineage Slingshot pseudotime trajectory was constructed using all principal components, with the FACS-sorted PLT population being the start cluster and the MUL population being the end cluster. The resulting pseudotime values for each cell were then used to infer their molecular proximity relative to these two reference populations.

Poly(I-C) treatment

Mice were treated with poly(I-C) as described (Bogeska et al., 2022). Mice were injected a total of 8 times i.p. with high molecular weight poly(I-C), two injections per week for four weeks, followed by a four-week recovery period prior to bone marrow harvest and isolation of HSCs via flow cytometry.

Romiplostim treatment

Romiplostim (Nplate) powder was reconstituted to a 500 μg/mL stock solution according to the manufacturer's instructions and further diluted in PBS to a 10 μg/mL working concentration. Mice were administered Romiplostim or PBS at 10 μL/g body weight via subcutaneous injection on days 1, 5, 8, and 12 (twice weekly for two weeks). Peripheral blood was collected from the tail vein on days 0 (baseline), 7, and 14 for platelet count measurements. Bone marrow was harvested at the experimental endpoint on day 15.

Anti-CD42b antibody platelet depletion

Anti-CD42b antibody (Emfret, R300) and control antibody (non-immune rat IgG, Emfret, C301) were administered as a single dose at 2 μg/g body weight via intravenous injection. Peripheral platelet counts were measured at baseline and 24 h post-injection, at which time animals were sacrificed, and bone marrow samples were collected for downstream analyses.

Candidate CITEseq and Fluidigm marker identification

For the HSC clone bulk RNAseq data, DESeq2 (Love et al., 2014) was used to perform differential analysis ($p < 0.05$, log2FC > 0.5) between paired HSC subpopulations: Platelet-restricted (P), Platelet-Erythrocytes-Myeloid-restricted (PEM) and Multi-lineage (MULTI). Differentially expressed genes were filtered for based on the following criteria: TPM >5 in at least one clone, maximum TPM variability <200 between clones of the same lineage reconstitution pattern. For CITEseq candidates, localisation to the cell membrane (integral component of plasma membrane (GO:0005887)) and the availability of a FACS antibody were also required.

Single-cell multiome data processing

Raw FASTQ data was demultiplexed using Cellranger-arc (10×) with the mm10 reference genome to generate RNA and ATACseq feature-by-barcode count matrices; the pipeline also performs joint-modality cell calling that identifies single-cell associated barcodes. The CITEseq data were demultiplexed using CITE-seq-Count (Grieshaber-Bouyer et al., 2021). Count data from different modalities were combined using Seurat v5 (Hao et al., 2024). For each cell, the nucleosome signal, TSS enrichment score, mitochondrial RNA gene percentage, and ATACseq blacklist region (Amemiya et al., 2019) fragment percentage were calculated. Cells were filtered based on the following criteria: ATAC peak region fragments >3,000, percentage of ATAC reads in peaks >15%, ATAC blacklist reads <5%, nucleosome signal <4, TSS enrichment score >2, and mitochondrial RNA gene percentage <15%. RNA expression data was log-normalised and scaled using Seurat. Antibody-derived tag (ADT) counts were normalised using centered log-ratio transformation. ATAC peak counts were normalised using term frequency-inverse document frequency (TF-IDF), followed by singular value decomposition (SVD). Cell cycle phases were determined by the Seurat CellCycleScoring function using published signatures (Tirosh et al., 2016). The motif accessibility was calculated using the RunChromVar function in Signac.

WNN clustering and dimensional reduction

Dimensional reductions were performed on the normalised RNAseq and ATACseq data. For the RNAseq data, JackStraw scores were calculated for principal component analysis (PCA) results to select the best dimensions. For the ATACseq data, dimensions were calculated using latent semantic indexing (LSI), and the correlation between the total counts and LSI-reduced dimensions was computed. To perform RNA+ATACseq joint analysis, the weighted nearest neighbor (WNN) (Hao et al., 2021) was computed using the appropriate dimensions from both modalities; unsupervised clusters and a uniform manifold approximation and projection (UMAP) were then generated.

In silico HSC subtype scoring

Signature gene and chromatin regions of platelet-restricted and multi-lineage clones were previously described (Meng et al., 2023). Single HSCs were scored for platelet (P) and multi-lineage (MULTI) potential using the bulk sequencing signatures. ATAC scores were calculated using ChromVAR (Schep et al., 2017) that counts the number of fragments within a given chromatin peak list. Per-cell lineage potential scores were calculated by dividing the PLT score by MULTI score in each modality, a higher lineage potential score indicates more platelet-bias and vice versa.

In silico marker selection and gating

Recursive feature elimination was used to rank the importance of CITEseq markers for separating the two extreme clusters, PLT and MUL. The top five ranked CITEseq markers were further validated: the HSCs were filtered by their CITEseq counts to simulated FACS enrichment of platelet-biased or multi-lineage phenotype.

Multiome data label transfer

To repeat the marker-based in silico gating analysis in aged HSCs, we transferred cluster labels from the 8-week reference dataset to 24-month-old HSCs using Seurat's anchor-based multi-omic framework. First, we computed a supervised PCA (sPCA) reduction on the RNA assay of the reference dataset, using the multimodal WNN graph to capture joint transcriptional and chromatin accessibility structure underlying the reference clustering. Based on this sPCA space, a neighbor graph was generated and used as the basis for transfer anchor identification between 8-week and 24-month HSCs with FindTransferAnchors. Cluster identities from the 8-week dataset were then transferred to the 24-month cells using MapQuery. To confirm that these transferred annotations were consistent with the intrinsic structure of the 24-month dataset, we also generated an independent WNN UMAP and visualised transferred labels. This allowed us to assess whether the predicted labels formed coherent groups within the dataset in its own multimodal manifold.

QUANTIFICATION AND STATISTICAL ANALYSIS

Multiome data analysis

Wilcoxon test was used to compare accessible chromatin sites. Differentially expressed genes or chromatin sites with log2FC > 0.2, adjusted $p < 0.05$ and minimal detection >10% in a cell population were selected. For expression data, MAST (Finak et al., 2015) was used to identify differentially expressed genes. GSEA analyses were subsequently performed using clusterProfiler (Yu et al., 2012).

Figure-level statistical analyses

Statistical tests for figure-level analyses were reported in the corresponding figure legends. In silico gating comparisons in Figures 2G and 4E were performed using chi-square tests. Differential gene analysis in Figure 3B was performed using DESeq2 Wald's test, and transplanted HSC lineage-output comparisons in Figures 3G and 3H were performed using chi-square tests. For Figure 5, HSC subtype staining was analyzed using multivariate analyses (MANOVA), and lineage bias scores were compared using unpaired two-tailed T-tests. For Figure 6, platelet counts were compared using unpaired two-tailed T-tests, and cell cycle analysis and FACS gating were analyzed using MANOVA. For supplementary analyses, Figure S3 used MAST for single-cell differential gene analysis and permutation tests for GSEA enrichment, and Figure S6 used unpaired two-tailed T-tests. Data are presented as mean ± standard deviation where indicated in the figure legends; significance thresholds are shown in the corresponding legends (*$p < 0.05$, **$p < 0.005$, ***$p < 0.001$).

Stem Cell Reports, Volume *21*

Supplemental Information

Multiome-based identification of molecular markers for prospective identification of platelet-biased HSCs

Bowen Zhang, Yiran Meng, Esther Rodríguez Correa, Xiying Ren, Alexandre Fagnan, Michael D. Milsom, and Claus Nerlov

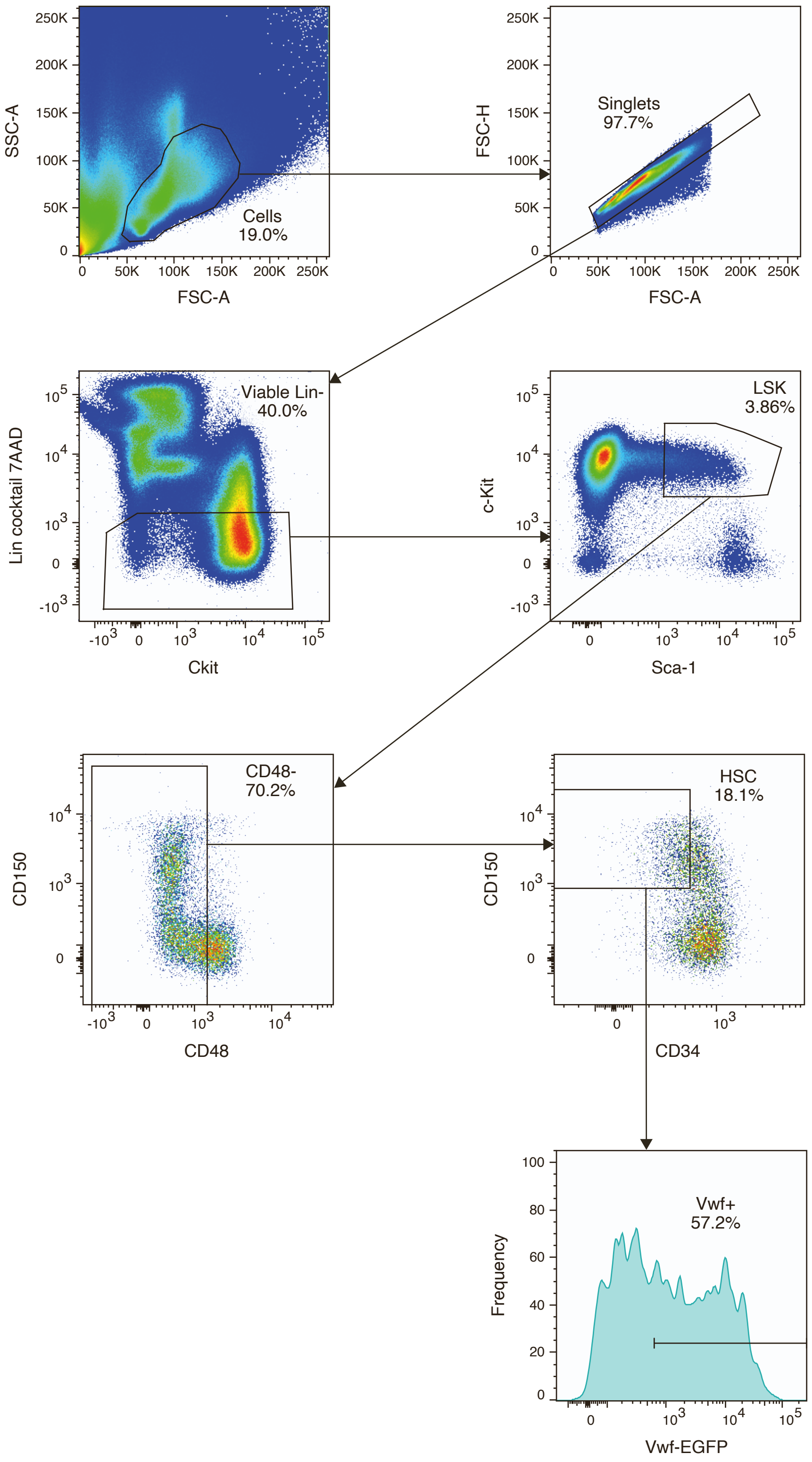

Supplementary Figure 1

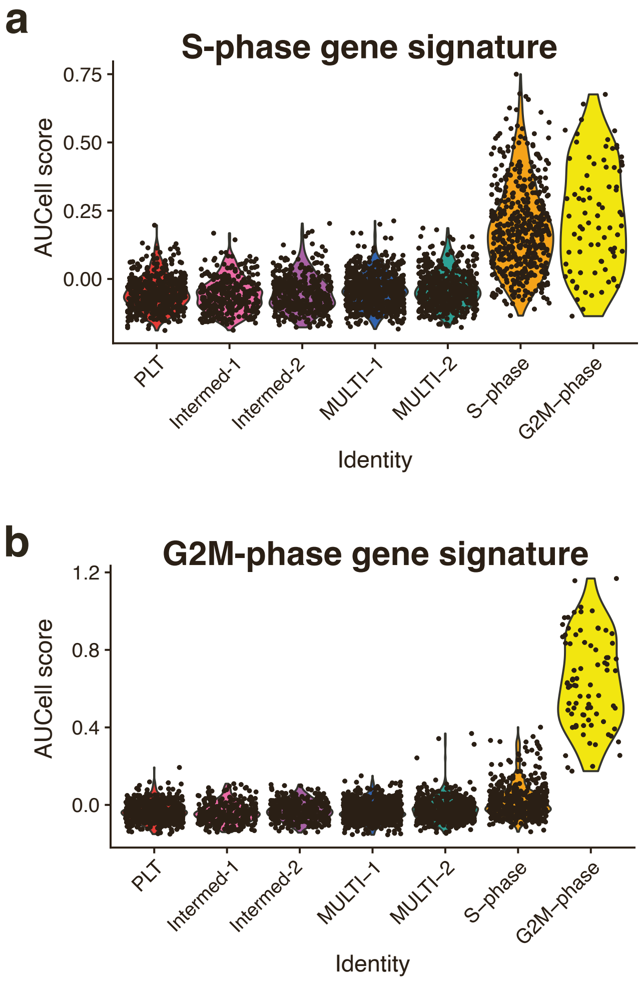

Supplementary Figure 2

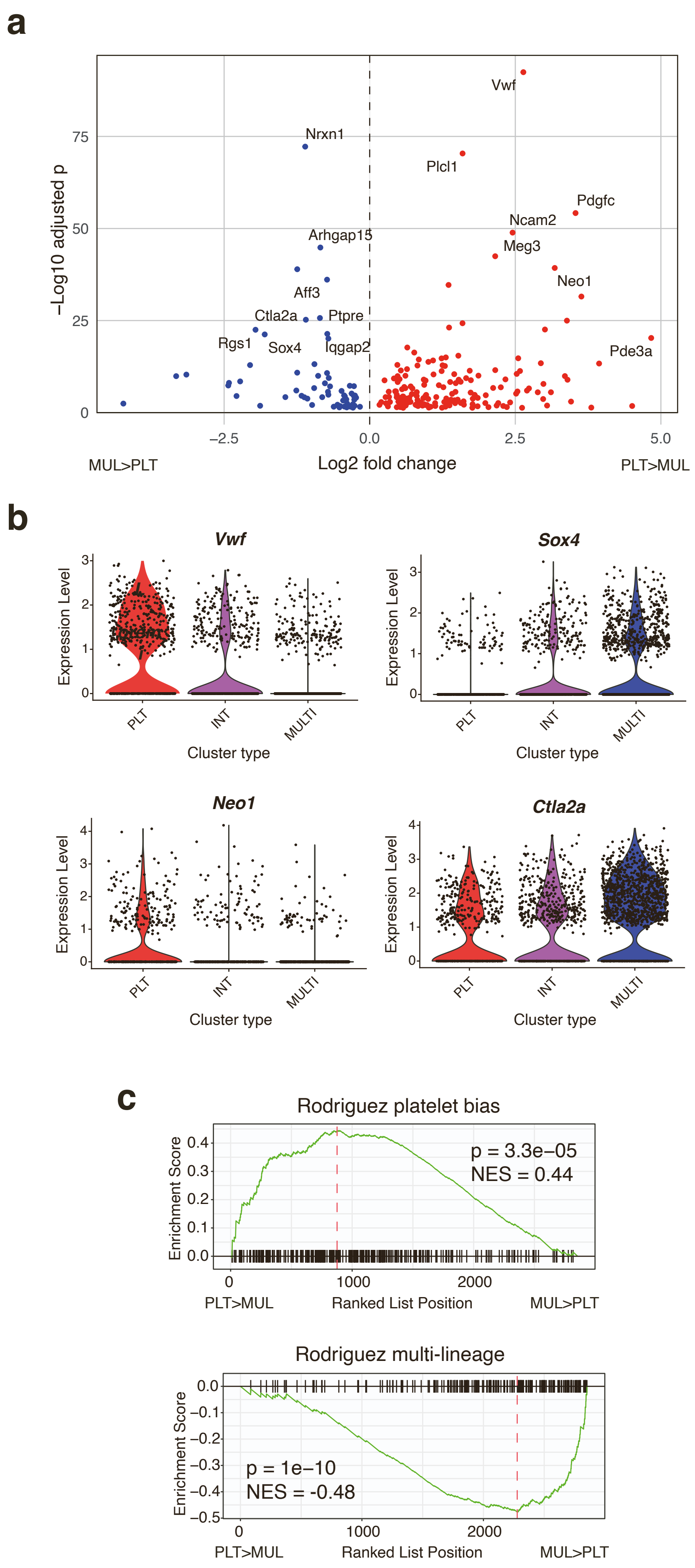

Supplementary Figure 3

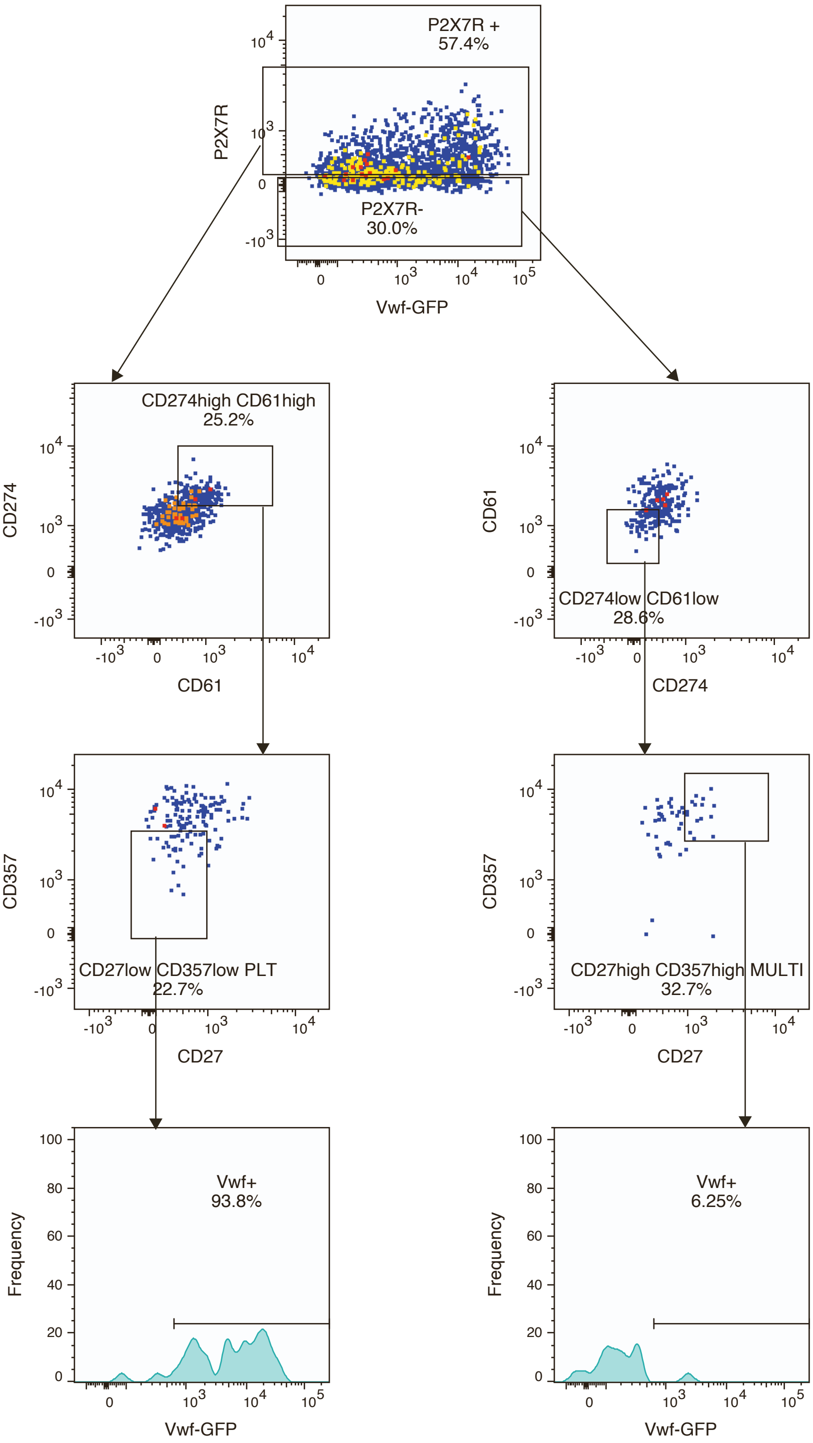

Supplementary Figure 4

a

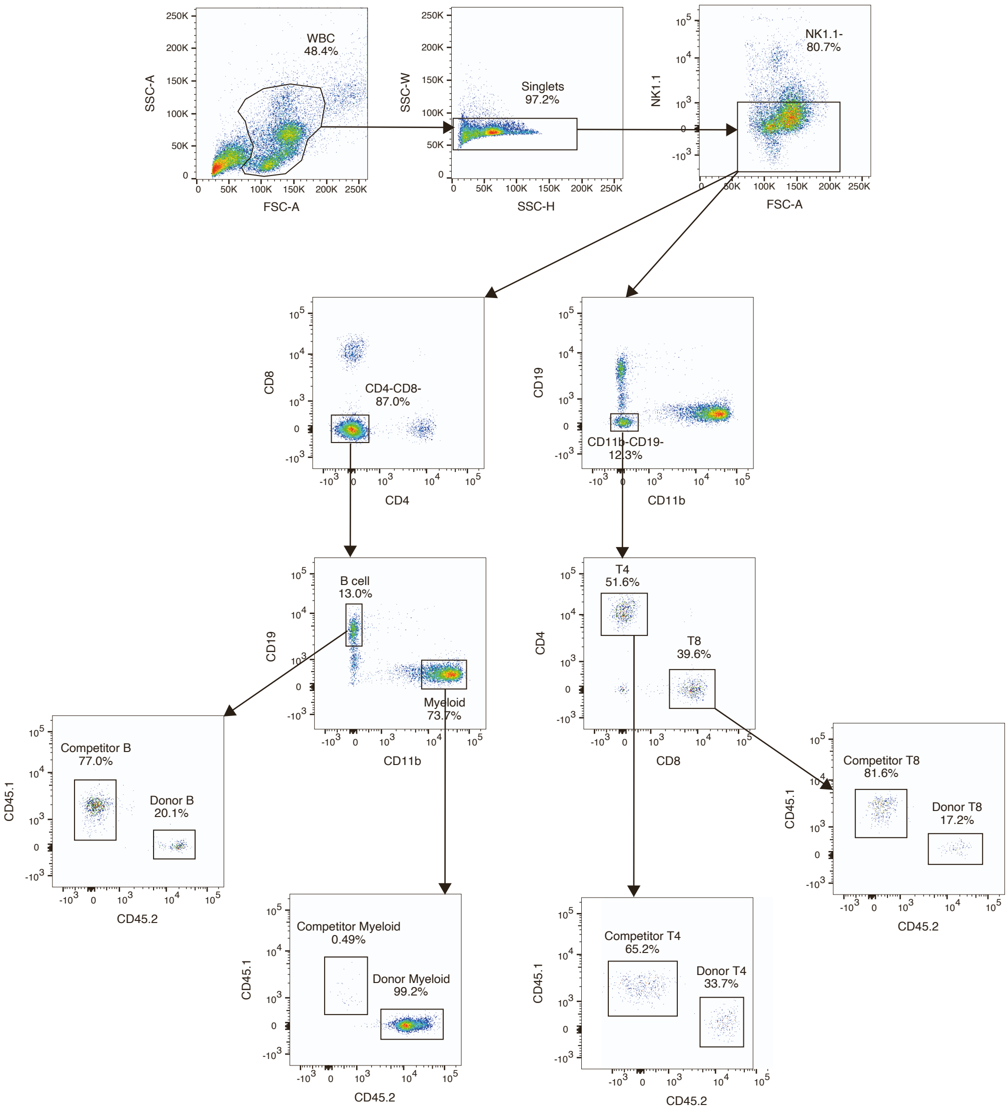

b

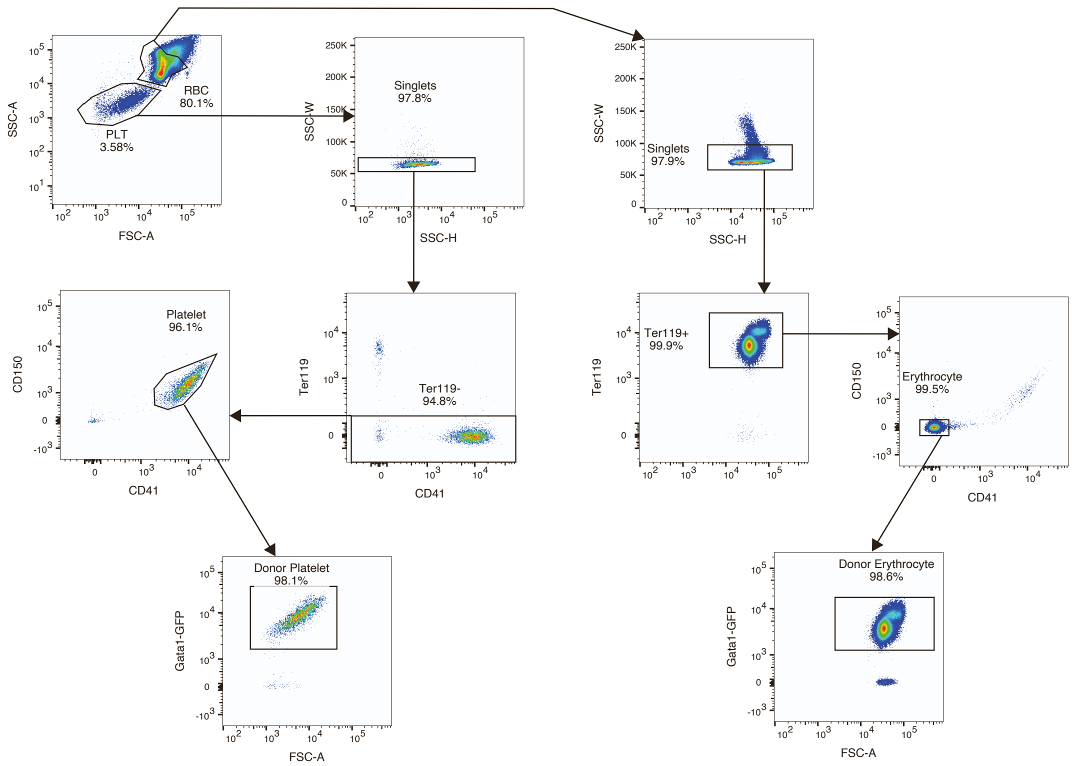

Supplementary Figure 5

a

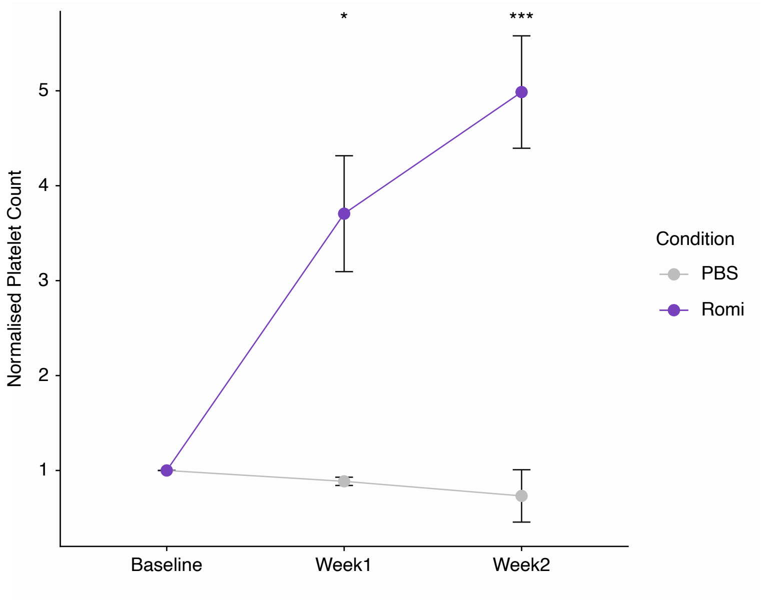

b

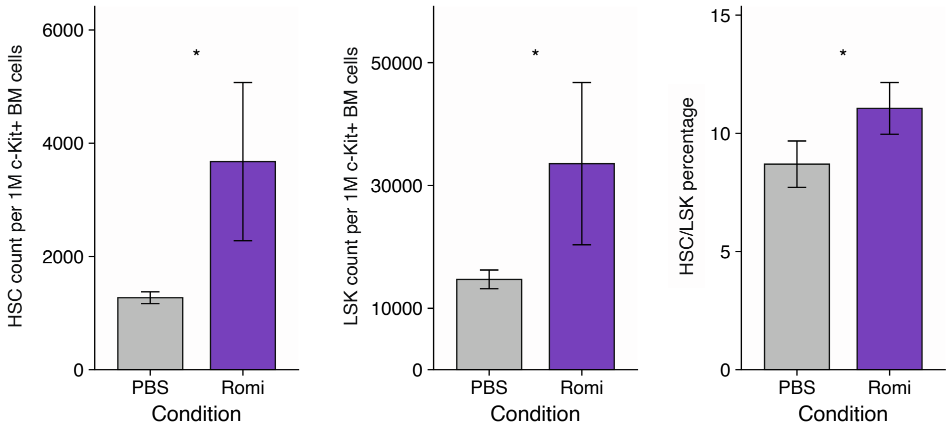

Supplementary Figure 6

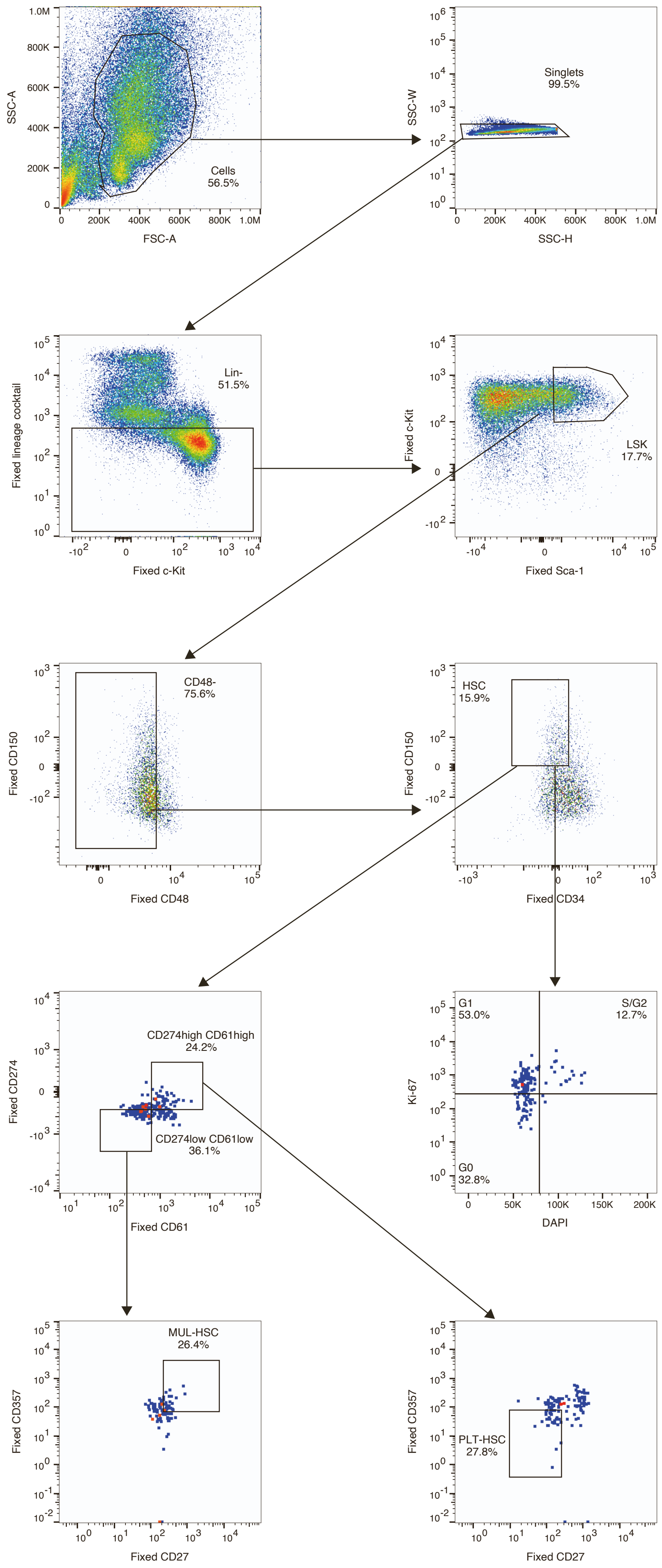

Supplementary Figure 7

Supplementary Figure legends.

Supplementary Figure 1 | Gating strategy for identifying mouse HSCs

Flow cytometry gating strategy for identifying HSCs (LSKCD150+CD48–CD34–) from *Vwf*-EGFP transgenic mice. The percentage of *Vwf*-EGFP positive cells in the total HSC population is shown. This strategy was also used for purifying HSCs from WT and *Gata1*-EGFP transgenic mice.

Supplementary Figure 2 | Cell cycling scores of 8-week HSCs.

A) Gene expression-inferred S phase score of clusters from Figure 2A.

B) Gene expression-inferred G2M phase score of clusters from Figure 2A.

Supplementary Figure 3 | Annotation of non-cycling WNN clusters

A) Volcano plot of genes differentially expressed ($P<0.05$) between PLT and MUL clusters in HSCs from Figure 2A.

B) Expression levels of selected lineage potential signature genes in the PLT, INT and MUL clusters from Figure 2A.

C) GSEA analysis comparing the PLT and MUL clusters using previously defined HSC lineage bias signatures for platelet-biased and multi-lineage HSCs.

Statistical comparisons performed using MAST for single-cell differential gene analysis and permutation test for GSEA enrichment.

Supplementary Figure 4 | FACS enrichment for *Vwf*+ HSCs with the five-marker strategy

Flow cytometry gating strategy using the top five RFE-ranked markers from Figure 2E. The percentage of *Vwf*-EGFP positive cells is shown in PLT- (LSKCD150+CD48–CD34–P2X7R+$CD61^{hi}CD274^{hi}CD357^{lo}CD27^{lo}$) and MUL-HSCs (LSKCD150+CD48–CD34–P2X7R-$CD61^{lo}CD274^{lo}CD357^{hi}CD27^{hi}$) from *Vwf*-EGFP transgenic mice.

Supplementary Figure 5 | Peripheral blood lineage reconstitution analysis in mice transplanted with single HSCs

A) Flow cytometry gating strategy for identifying donor-derived CD45.1 myeloid cells, B cells and T cells.

B) Flow cytometry gating strategy for identifying donor-derived *Gata1*-EGFP+ platelets and erythrocytes.

Supplementary Figure 6 | Peripheral blood and endpoint bone marrow analysis in mice treated with Romiplostim

A) Normalised peripheral blood platelet count after Romiplostim (Romi) treatment or control.

B) LSK and HSC (LSKCD150+CD48–CD34–) cell counts per million c-Kit-enriched bone marrow cells and the HSC/LSK ratio.

Mouse number: Romi, N=4; PBS, N=4. Statistical comparisons performed using unpaired two-tailed T-tests; *$P < 0.05$, ***$P < 0.001$. Data are presented as mean ± standard deviation.

Supplementary Figure 7 | Gating strategy for fixed mouse bone marrow

Flow cytometry gating strategy for identifying PLT- and MUL-HSC, and assessing cell cycle status in permeabilized mouse bone marrow samples. Cell cycle phases are defined as G0 ($DAPI^{lo}Ki\text{-}67^{lo}$), G1 ($DAPI^{lo}Ki\text{-}67^{hi}$) and S/G2/M ($DAPI^{hi}Ki\text{-}67^{hi}$)

<u>Supplementary Table legends.</u>

Table S1 | Differentially expressed genes between PLT and MUL-HSC clusters from 8-week-old mice.

Differential expression analysis was performed using MAST, with a significance threshold of $P < 0.05$.

Table S2 | Differentially expressed genes between clonal PLT and MUL-HSC populations.

Differential expression analysis was performed using DESeq2, with a significance threshold of $P < 0.0005$. The max_tpm column reports the highest transcript per million (TPM) values

detected across all clones. The var_PLT and var_MUL columns represent the maximum variation in TPM between biological replicates within the PLT and MUL clones, respectively.

Table S3 | Antibody-derived tags.

The titration column indicates the titration factor relative to the corresponding unconjugated antibody.

Table S4 | Oligos.

Shows the sequence of pre-amplification, sample index PCR primers for constructing DOGMAseq libraries, and the antibody derived tag oligo.

Table S5 | TaqMan assays for microfluidics-based qRT-PCR.

This panel allows accurate phenotyping of lineage potential in mouse HSCs.

Table S6 | Normalized gene expression values from microfluidics-based qRT-PCR of HSC populations.

Shows the integrated lineage potential signature gene expression profiles used in (Figure 5D).

Table S7 | FACS antibodies.

List of antibodies used for flow cytometry and cell sorting.
